# Exploring Diffusion and Aggregation Behaviors in Carbohydrate Solutions

**DOI:** 10.1021/acs.jpcb.5c07628

**Published:** 2026-01-08

**Authors:** Samuel G. Holmes, Sawsan Mahmoud, Robert J. Woods

**Affiliations:** Complex Carbohydrate Research Center, 1355 University of Georgia, Athens, Georgia 30602, United States

## Abstract

Engineered glycomaterials represent an exciting new field of biomaterials, owing to their vast structural diversity, yielding a myriad of potential properties and applications. Glycomaterials can be composed of naturally occurring polysaccharides (cellulose, hyaluronic acid, chondroitin sulfate, etc.), but these are also amenable to chemical derivatization, resulting in engineered glycomaterials with altered chemical and material properties. However, rules for predicting the properties of glycomaterials, based on their chemical structure, are not well established, hindering their rational design. Computational methods, such as molecular dynamics (MD) simulation, can accurately characterize the spatial and temporal properties, of glycomaterials; however, the application of MD simulations to predict material properties, such as diffusion, solubility, viscosity, and hydrogel formation, has received less attention. This work demonstrates that diffusion properties of well-known glycomaterial constituents, measured by DOSY NMR spectroscopy and calculated from explicit solvent MD simulations with the GLYCAM06 force field, generally agree well. However, the theoretical results are found to be heavily dependent on the water model, with the TIP5P and OPC models outperforming the widely used TIP3P model. Lastly, an empirical method for estimating the diffusion properties of carbohydrates, based on assessing the number of tightly bound waters, is proposed. Together, these results illustrate the potential of computational approaches to guide the rational design of engineered glycomaterials.

## Graphical abstract

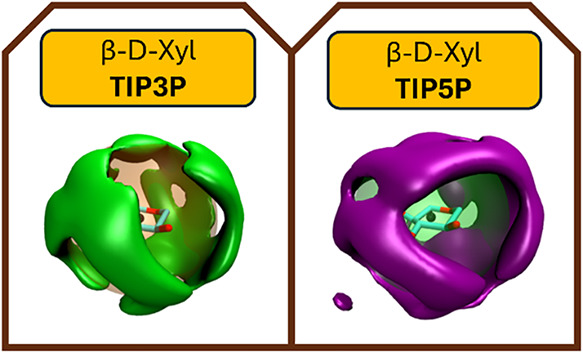

## Introduction

Engineered glycomaterials represent an exciting application of biomaterials science, having found widespread uses in biomedical science and industry.^1–5^ Glycomaterials largely fall into two categories based on their polysaccharide chemical structure: those that are naturally occurring (cellulose, starch, xylan, hyaluronic acid, chondroitin sulfate, etc.) or those that are created by the chemical modification of a natural polysaccharide, for example, by acetylation, alkylation, sulfation, carboxylation, etc. (Figure 1). At present, rules for predicting the material properties (such as the diffusion coefficient (*D*) and hydrodynamic radius (*R*_H_)) of natural or engineered glycomaterials are not well established, hindering their rational design.

**Figure 1:**
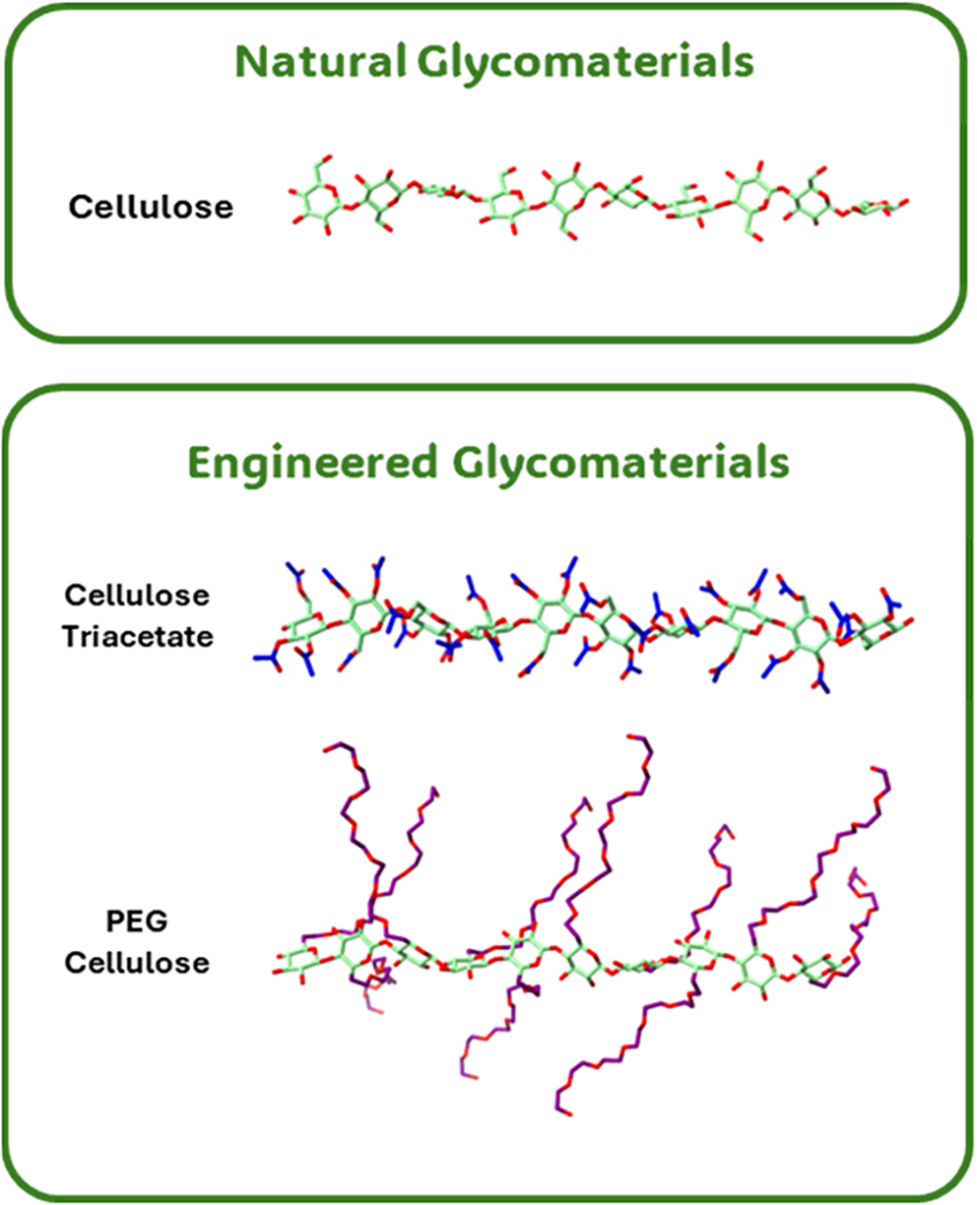
Examples of glycomaterials and their 3D structures. Glycomaterials can be either natural or engineered, with the latter typically comprising a carbohydrate backbone that has been chemically modified. The examples above contain a cellulose decasaccharide backbone (upper) with acetate and polyethylene glycol (PEG) groups shown in blue and purple, respectively (lower), carbohydrate carbon atoms are shown in green, and oxygen atoms in red.

The experimental determination of *D* for glycomaterials is relatively straightforward;^6–11^ however, establishing structure–property relationships that govern its value requires a deeper understanding of the physical properties of solute–solvent interactions that impact its solution behavior. Such relationships are crucial for rationally designing glycomaterials, a capability that would greatly amplify the utility of readily available polysaccharides. Computational approaches to modeling diffusion properties have several potential benefits, including the ability to be employed prospectively, which could potentially guide the development of novel materials and the creation and testing of structure–function hypotheses. Here, we critically evaluate the ability of MD simulations to accurately predict carbohydrate diffusion and aggregation properties, employing the GLYCAM06 force field for carbohydrates,^12^ and three popular water models.

Explicit solvent MD simulations provide an attractive alternative to experimental approaches for determining *D*,^13–16^ particularly because they can be employed prospectively to guide the synthesis of novel materials. Calculations of diffusion properties are currently limited to relatively small polymers (comprising fewer than about 50 monosaccharides) because of the computational demand required to achieve configurational convergence.^17,18^ Nevertheless, MD simulations can be performed on virtually any glycomaterial for which the appropriate force field parameters exist, which is ideal for screening libraries of glycomaterials and developing structure–property relationships. Furthermore, a wide variety of atomic and molecular-level descriptors can be calculated from MD simulations and related to *D*, such as solvent-accessible surface area, hydrophobicity, inter- and intramolecular hydrogen bonding, solvation structure, and three-dimensional (3D) shape, which are challenging if not impossible to obtain by experiment.

All common biomolecular force fields include the capability of simulating carbohydrates;^19^ however, there has been relatively little attention given to predicting carbohydrate diffusion properties, and most such studies have focused on small common carbohydrates, such as glucose, sucrose, or trehalose.^20–22^ Additionally, as seen from the Stokes–Einstein relationship (eq 1), *D* depends inversely on solvent viscosity, which varies considerably between common theoretical water models.^23^

1 $$D=\frac{k_{B}T}{6\pi \eta R_{H}}$$

where, *k*_B_ is the Boltzmann constant, *T* is the temperature, η is the viscosity of the solvent, and *R*_H_ is the hydrodynamic radius of the solute.

Thus, the accurate derivation of *D* by MD simulation depends on both the solute and solvent force fields and on the relative balance of inter- and intramolecular forces. As a prerequisite for the validation of theoretical predictions of *D*, we first established an experimental reference set of values, using DOSY NMR, for 18 carbohydrates, varying in composition from monosaccharide to heptasaccharide (Table S1). Each of the carbohydrates was also subjected to MD simulations with the GLYCAM06 force field and the data evaluated with an emphasis on quantifying the influence of solvent model on diffusion properties for three common water models (TIP3P,^24^ TIP5P,^25^ and OPC^26^). The results were interpreted in terms of the impact of viscosity on carbohydrate diffusion and on the extent to which each water model promoted carbohydrate aggregation. Additionally, radial distribution functions (RDFs) and water occupancy maps were employed to assess the extent to which the water structure proximal to the carbohydrate was sensitive to the water model and might have impacted the computed diffusion properties. Finally, an approximation was introduced that enabled estimation of the diffusion coefficient (*D*) of an oligosaccharide by approximating its hydrodynamic radius, based on a knowledge of its radius of gyration (*R*_g_) and the number of entrained waters.

## Methods

### NMR Experiments

The diffusion coefficients of all carbohydrates in Table S1 were measured by DOSY NMR. All carbohydrates, with the exception of *methyl 2,3,4,6-tetra-O-acetyl-a-* d *-glucopyranoside* (Tetra–O-Ac-Glc-α-OMe), were available for purchase from Sigma-Aldrich (Burlington, MA), Thermo Fisher Scientific (Waltham, MA), or Combi-Blocks (San Diego, CA). Each carbohydrate was resuspended at a concentration of 50 mM in 95% nanopure water (600 uL) with 5% D_2_O added for signal locking. A ratio of 95:5 H_2_O:D2O was used as the NMR solvent instead of pure D_2_O because diffusion is not identical in D_2_O vs H_2_O, due to slight differences in hydrogen bond strengths.^27,28^ Diffusion coefficients were measured on a Varian 600 MHz NMR spectrometer at 298 K from triplicate samples. All carbohydrates were soluble at 50 mM except for Tetra–O-Ac-Glc-α-OMe, whose diffusion coefficient was measured at 22 mM. A concentration of 50 mM was chosen to improve the efficiency of the MD simulations, which become very long when the simulation cell becomes large enough to reach lower concentrations, and also to enhance the sensitivity of NMR experiments, which required at least a mM concentration at a field strength of 600 MHz. NMR spectra were acquired by using Bruker Topspin software. The “steppesgp1s” pulse sequence was used for DOSY experiments, which features a water suppression pulse using excitation sculpting and bipolar gradient pulses. For each measurement, 32 gradient strengths were employed using a quadratic ramp shape. The time between gradient pulses (Δ) and the length of the gradient pulse (δ) were 60 and 2 ms, respectively, and a 3 s delay was used between subsequent scans. For select carbohydrates, concentrations of 5 and 25 mM were also prepared so that infinite diffusion coefficients could be estimated via extrapolation.

### Simulation Protocol

Initial 3D structures of the carbohydrates were built using the carbohydrate builder using the GLYCAM-Web server (www.glycam.org/cb), including both anomers for each reducing sugar. To compare simulated and experimental diffusion coefficients at the same concentration, each system was prepared using PACKMOL.^29^ Twenty identical copies of each carbohydrate were distributed in a cubic simulation cell with a volume of 664,424 Å^3^, resulting in a carbohydrate concentration of 50 mM. Water molecules were added to fill the remaining space in the cell at the density of pure water. The number of waters added to each system ranged from approximately 21,500 to 22,200. Counter ions were added to charge-balance the system as needed. Simulations were carried out using the pmemd simulation engine in AMBER21. A time step of 2 fs was used during simulations and the nonbonded cutoff distance was set to 8.0 Å. The SHAKE algorithm was enabled to constrain all hydrogen-containing bond lengths. Although use of an NPT ensemble is standard for most applications of MD simulations, it is not appropriate for predicting transport properties.^30^ Therefore, MD simulations were performed in the NVE ensemble. To achieve an equilibrated NVE ensemble, the following multistep protocol was employed. First, each system was subjected to a minimization, equilibration, and NPT production phase (200 ps) to equilibrate density using a standard 10-step protocol.^31^ The densities of all systems studied equilibrated in the production NPT phase within 50 ps (Figure S1). Next, a frame within the 50–200 ps window from the production NPT was selected as the starting point for the production NVE, such that the volume of the simulation cell from that frame was within 3 Å^3^ of the average volume of the NPT run. This served to minimize the drift in average total energy, temperature, and pressure in the production NVE phase after removing the barostat and thermostat. Minimal drifts in average total energy and temperature were observed (Figures S2 and S3). Thus, this simulation protocol was able to generate stable trajectories in the NVE ensemble for determinations of *D*. Each NVE system was subjected to production MD simulations for 10 ns, with trajectories saved every 10 ps. Ten replicate simulations were performed to calculate *D*, resulting in a total of 2 μs of simulation time per carbohydrate. In addition, to evaluate the effects of concentration on *D*, 200 simulations (10 ns) of a single carbohydrate in water were performed with an identical system size to the 50 mM simulations (664,424 Å^3^), which mimicked infinite dilution conditions (Figure S4).

### Calculation of Diffusion Coefficients

*D* values were derived from the slope of the plot of the mean squared displacement ⟨Δ*r* ^2^(*t*)⟩ of the carbohydrate vs simulation time (*t*) (eqs 2 and 3).

2 $$D=\frac{\left\langle \Delta r^{2}\left(t\right)\right\rangle }{2\mathrm{nt}}$$

where *n* is the number of dimensions (3) over which ⟨Δ*r* ^2^(*t*)⟩ was computed (eq 3) and

3 $$\Delta r^{2}\left(t\right)=\frac{1}{N}\sum_{i=1}^{N}{\left(\sqrt{{\left(x_{i}\left(t\right)-x_{i}\left(0\right)\right)}^{2}+{\left(y_{i}\left(t\right)-y_{i}\left(0\right)\right)}^{2}+{\left(z_{i}\left(t\right)-z_{i}\left(0\right)\right)}^{2}}\right)}^{2}$$

where the coordinates are relative to the center of mass of the carbohydrate and *N* is the total number of solutes (20) per simulation. Ensemble averaging (20 solutes per periodic box) was performed at 50 mM to match the conditions of the NMR experiment, and had the advantage of increasing the efficiency of the calculation.^32^ The mean squared displacement was averaged over 10 replicate simulations at each time point, with *D* calculated via linear regression (Figure 2).

**Figure 2:**
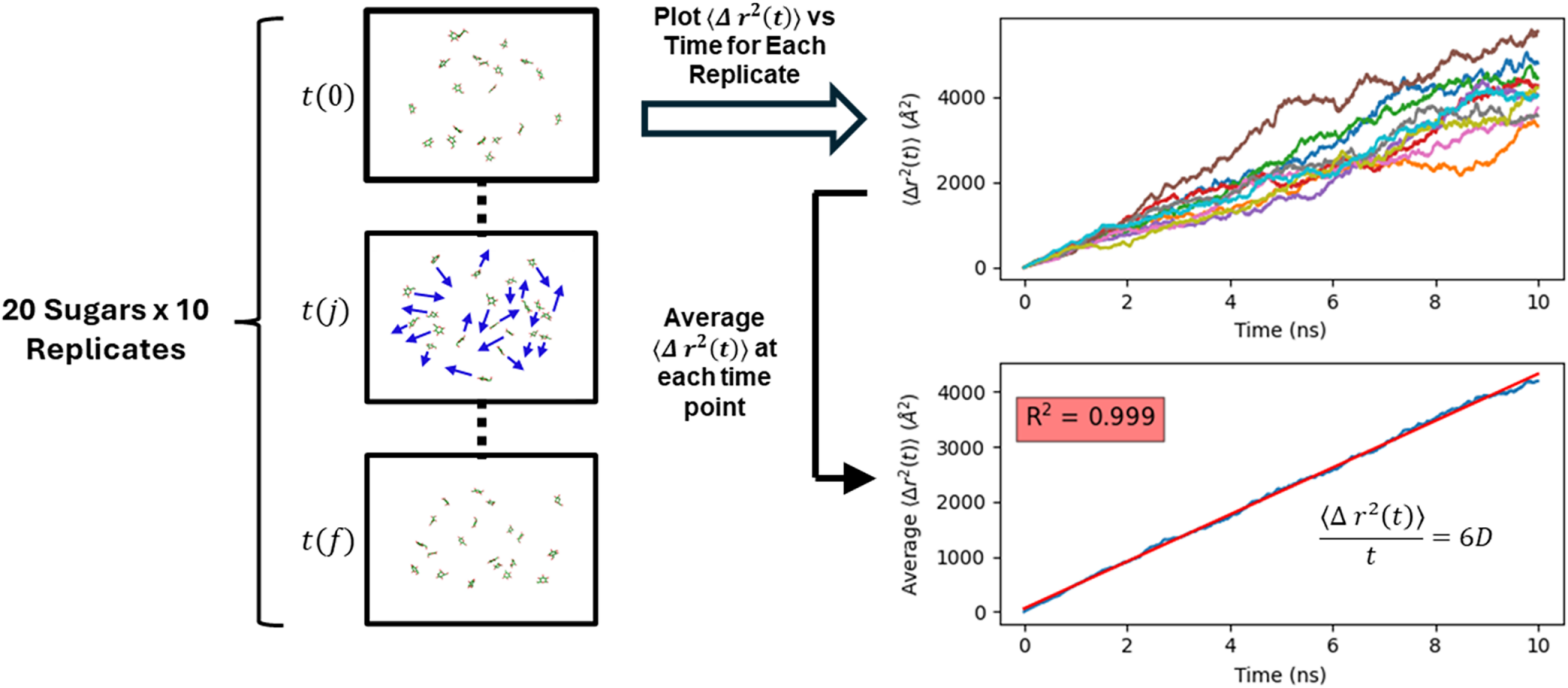
Schematic protocol for deriving *D* by MD simulation, where *t­(0)* and *t­(f)* represent the initial and final time points of the simulation, respectively. *D* is obtained from the slope (6*D*) of the linear fit to the average of (Δ*r* ^2^(*t*)), red line, lower right panel.

### Calculation of *R*_g_ and *R*_H_

Hydrodynamic radii (*R*_H_) were computed (eq 1) at 298 K. Radii of gyration (*R*_g_) were calculated by using the “radgyr” command in CPPTRAJ.

### Statistical Analysis

For comparisons of two means, a two-tailed Welch’s *t* test was used to account for unequal variances. For comparisons of more than two means, a one-way ANOVA was performed to test for differences between the groups. When the ANOVA indicated a statistically significant difference, Tukey’s Honestly Significant Difference (HSD) test was used as a *post hoc* procedure to identify which specific pairs differed, while controlling for the family wise error rates. Statistical significance was assessed at an α level (*p*-value) of <0.05 unless otherwise noted.

### Radial Distribution Functions and Water Occupancy Maps

The average number of waters in the first solvation shell around the solute, commonly referred to as *N*_1_,^33,34^ was calculated from the radial distribution function (RDF, *g­(r)*, eq 4).

4 $$N_{1}=4\pi \rho \int_{0}^{r_{\min}}g\left(r\right)r^{2}dr$$

where ρ is the number density of waters in the simulation and *r*_min_ is the bottom of the well between the first and second peaks in the RDF curve. The *g­(r)* function was calculated from the geometric center of the monosaccharide to all water oxygen atoms. Water occupancy maps were generated using the “volmap” command in CPPTRAJ^35^ with 0.25 Å spacing and a 25 Å cubic grid centered on the solute. Each voxel value corresponds to the time-averaged Gaussian-weighted probability density for observing a water oxygen atom in the voxel during the simulation.

### Synthesis of Methyl 2,3,4,6-Tetra-O-acetyl-α-d-glucopyranose (Tetra–O-Ac-Glc-α-OMe)

Methyl α-d-glucopyranoside (2 g, 10.30 mmol) purchased from Thermo Fisher Scientific was dissolved in excess acetyl chloride (20 mL) and stirred overnight at room temperature under argon. The solution was then evaporated under reduced pressure, and the residue was coevaporated with toluene (3 mL × 20 mL) to give a colorless syrup. The crude mixture was purified by CombiFlash chromatography using a dichloromethane:methanol solvent system, yielding the fully acetylated product (2.8 g, 75%) as a colorless syrup. ^1^H NMR (600 MHz, D_2_O) δ 5.43 (dd, *J* = 19.3, 9.6 Hz, 1H), 5.15 – 5.06 (m, 3H), 4.42 – 4.37 (m, 1H), 4.24 – 4.18 (m, 2H), 3.46 (s, 3H, OCH_3_), 2.13 (s, 3H, OAc), 2.10 (d, *J* = 5.5 Hz, 6H, 2 OAC), 2.07 (s, 3H, OAc).^13^C NMR (151 MHz, D_2_O) δ 173.76 (CO, Ac), 173.25 (CO, Ac), 172.80 (CO, Ac), 172.57 (CO, Ac), 96.46, 70.75, 70.31, 68.31, 66.93, 61.93 (C6), 55.19 (OCH_3_), 20.12 (2Ac-*C*H_3_), 20.09 (Ac-*C*H_3_), 20.05 (Ac-*C*H_3_). MS-ESI *m*/*z* [M + Na]^+^ Found 385.12. NMR spectra are included in the Supporting Information (Figure S5).

## Results and Discussion

### Effect of the Water Model

The diffusion of solutes in water is dictated by solute–solute (aggregation), solute–solvent (friction), and solvent–solvent (viscosity) interactions. Therefore, when deriving *D* from MD simulations, the choice of solute and solvent force fields can directly impact the computed value.^36,37^ Here, three popular water models (TIP3P, TIP5P, and OPC) were evaluated for their ability to predict the diffusion properties for the well-characterized carbohydrate d-glucopyranose (Glc) with the GLYCAM06 carbohydrate force field. The TIP3P model predicted *D* to be approximately 100% higher than the experimental value, consistent with its artificially low viscosity;^23^ in contrast, the OPC and TIP5P models led to near quantitative agreement with experiment (Figure 3). The values from OPC and TIP5P were statistically indistinguishable from each other, despite the fact that those two water models are structurally different (the former consisting of a tetrahedral arrangement of partial charges, and the latter planar).^26^ These results demonstrated that modest differences in the force field parameters of water models can profoundly impact not only bulk solvent properties but also solute transport properties.

**Figure 3:**
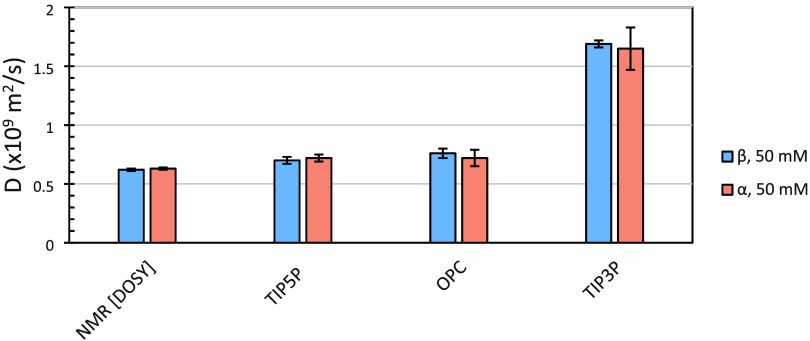
Effect of the water model on the calculated diffusion coefficient of the two anomers of Glc from explicit solvent MD simulations using the GLYCAM06 force field compared to experimental values.

Notably, no significant difference in *D* was observed between the two anomers of Glc determined either by MD simulation (regardless of the water model) or by DOSY NMR. The experimental values (α: *D* = 0.63 × 10^–9^ m^2^·s^–1^ ± 0.01, β: *D* = 0.62 × 10^–9^ m^2^·s^–1^ ± 0.01, *p* = 0.29) are in good agreement with the value of 0.63 × 10^–9^ m^2^·s^–1^ reported by Nagy et al.^38^ for both anomers. Interestingly, Yamanoi et al.^39^ reported that DOSY NMR gave rise to different *D* values for each anomer of Glc (α: 0.76 × 10^–9^ and β: 0.58 × 10^–9^ m^2^·s^–1^), and yet found that the *D* values for the p-hydroxyphenyl glycosides of Glc (α: 0.59 × 10^–9^ and β: 0.58 × 10^–9^ m^2^·s^–1^) showed almost no sensitivity to the anomeric configuration, a surprising result given the large size of the aromatic aglycon. None of the present data suggest a difference in the *D* values for either carbohydrate anomer (Figures 3 and S6).

To determine the extent to which each water model might also form different local interactions with the solute, radial distribution functions (RDFs) were computed from the MD data for simulations of β-Xyl (Figure 4). β-Xyl was selected for analysis because it lacks the exocyclic hydroxymethyl group present in Glc, and therefore the RDF would not be influenced by water-model-dependent differences in the rotamer populations of the exocyclic C5–C6 bond. Overall, the differences in solvation metrics derived from the RDF curves were subtle (Table 1). The maximum peak height in the TIP3P RDF was lower than those of the other models, but the difference was only weakly significant (*p* = 0.03), and the peak centers and second shell structures were statistically indistinguishable between water models. Interestingly, the number of waters in the first solvation shell (*N*_1_) was slightly higher in the OPC simulations than in TIP5P (*p* = 0.0001) or TIP3P (*p* = 0.0001), suggesting that, although the average thickness of the first solvation shell was similar across water models, the OPC model incorporated 1–2 additional waters in the first solvation shell relative to TIP5P or TIP3P.

**Figure 4:**
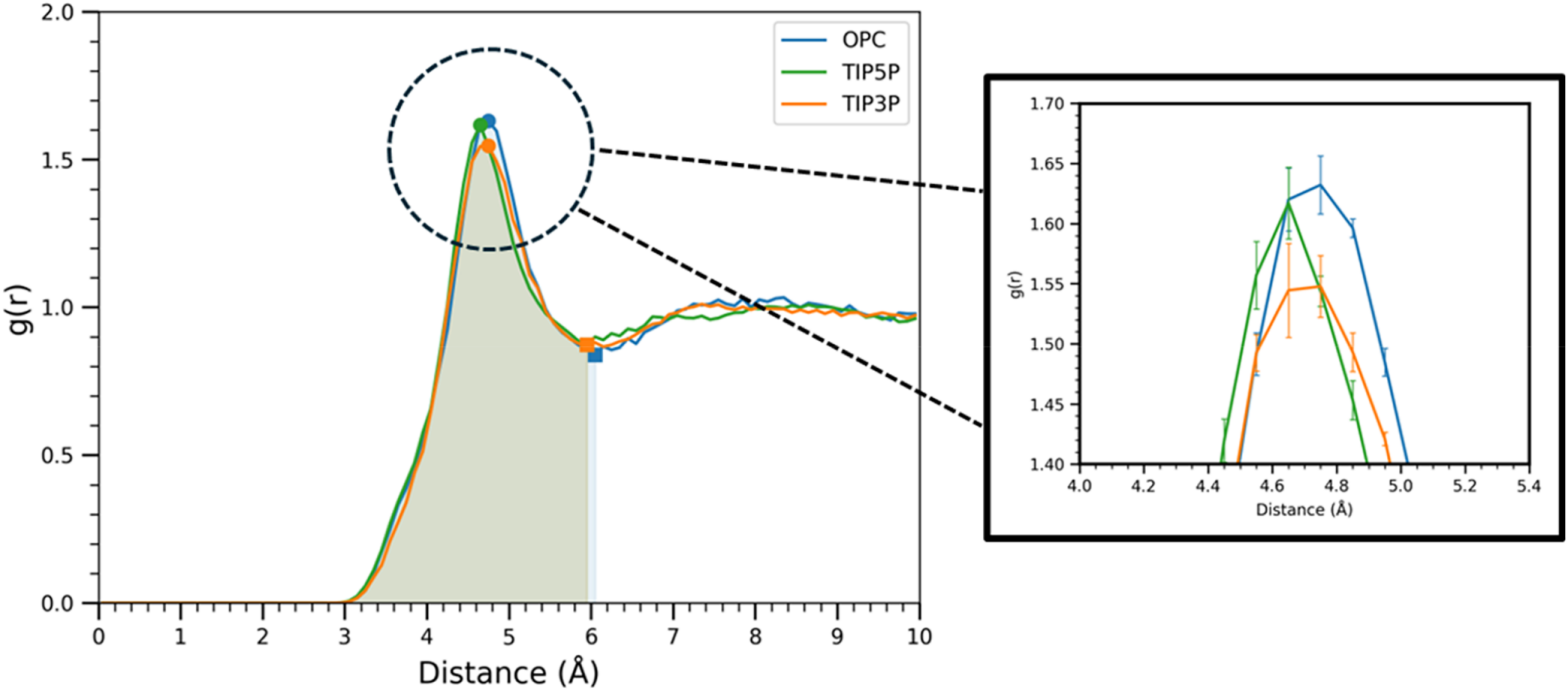
RDF curves for β-Xyl in three water models (TIP3P, TIP5P, and OPC) from 3 replicate simulations. Shaded regions indicate the first solvation shell area integrated to obtain *N*_1_. The right plot depicts the fine differences in peak heights of the first solvation peak in each water model, with standard deviations shown as error bars.

**Table 1:** RDF Statistics for Figure 4, Including the Center of the First Solvation Shell, Its Corresponding Maximum and Minimum, and the Average Number of Waters in the First Solvation Shell (*N*_*1*_)

	first peak position (Å)	RDF maximum	RDF minimum	*N*_1_
OPC	4.68 ± 0.06	1.63 ± 0.02	0.84 ± 0.02	26.02 ± 0.07
TIP5P	4.62 ± 0.06	1.62 ± 0.03	0.87 ± 0.01	24.40 ± 0.10
TIP3P	4.72 ± 0.06	1.56 ± 0.03	0.86 ± 0.01	24.27 ± 0.03

To probe the fine structure of water-carbohydrate interactions, spatial water occupancy maps were computed for β-Xyl. Although both the OPC and TIP5P models gave rise to comparable water structures at the carbohydrate surface in the vicinity of the first solvation shell, in contrast, the TIP3P interactions appeared less well-ordered (Figures 5 and S7). The water maps are similar to those determined previously from MD simulations of Glc,^40^ which found that increased water density was observed in the directions of hydroxyl group vectors (along the carbohydrate O–H bond directions) and the lone pair vectors of the carbohydrate oxygen atoms, consistent with the directionality of carbohydrate-water hydrogen bonds. While the weaker ordering of TIP3P water around the monosaccharide did not result in significantly fewer waters within the first solvation shell (Table 1).

**Figure 5:**
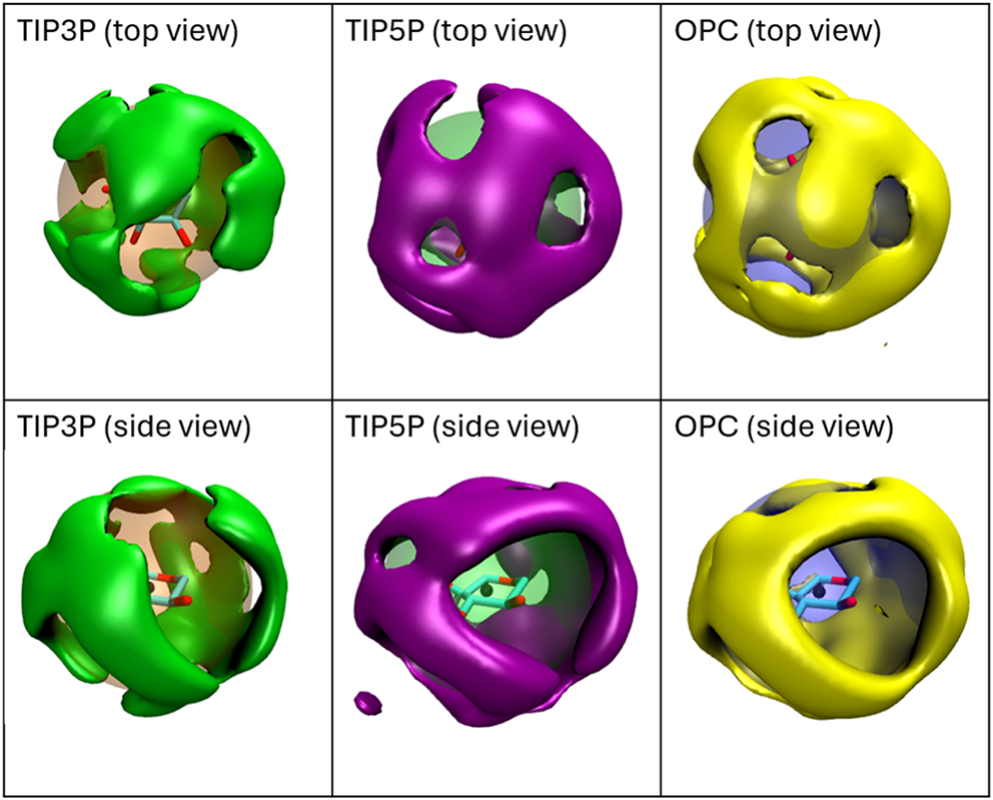
Water occupancy maps for the first solvation shell with a density cutoff (3.6%) chosen for optimal visualization; maps were also generated at cutoffs of 3.8% and 4.0% (Figure S7). The radii of the large translucent spheres (orange, green, and blue) correspond to the maximum peak height in the pertinent RDF curves (Figure 4) and provide reference for the location of the first solvation shell. The geometric center of the monosaccharide is indicated by a black sphere. Occupancy maps are displayed from the top of the monosaccharide (upper row) and from the side (lower row).

### Impact of Carbohydrate Size

Having identified OPC and TIP5P as optimal water models when paired with the GLYCAM06 force field, their performance was benchmarked against experimental *D* values for a range of 18 carbohydrates from mono- to heptasaccharides (Table S1). As expected,^41,42^ both experimental and theoretical *D* values displayed a strong dependence on solute molecular weight, with lower *M*_W_ solutes diffusing more rapidly (Figure 6). The smallest solute (Xyl) exhibited the largest experimental *D* (0.71 ± 0.01 × 10^–9^ m^2^/s, averaged over both anomers). Notably, simulations with the GLYCAM06 force field provided accurate predictions of carbohydrate *D* values, with either the TIP5P (mean absolute error (MAE) = 0.05 ± 0.04) or the OPC (MAE = 0.04 ± 0.03) water models (Table 6). As seen earlier for Glc, the *D* values for the reducing sugars in the data set showed no significant dependence on anomeric configuration (Figure S6).

**Figure 6:**
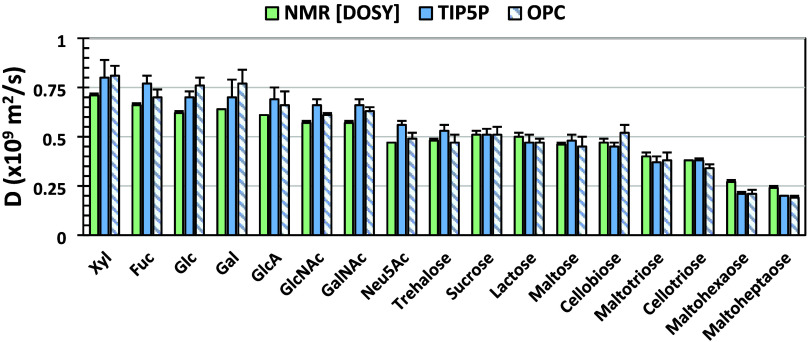
Experimental (green) and computed *D* values from MD simulations with the TIP5P (blue) or the OPC (blue/white stripe) water models at 50 mM carbohydrate concentrations. For reducing sugars, *D* is shown only for the β-anomer (see Figure S6 for both anomers).

### Concentration Effects

Although for most of the carbohydrates, the theoretical values for *D* were comparable to the experimental values, for the largest oligosaccharides (maltohexa- and -heptaose), the theoretical values modestly underestimated the experimental ones. To determine whether this behavior was concentration dependent, infinite dilution values from DOSY NMR experiments were obtained by extrapolation of the NMR data at 5, 25, and 50 mM solute concentrations for Glc and Maltoheptaose (Figure S8). At 50 mM, the experimentally determined *D* values for both the mono- and heptasaccharide were slightly lower than at infinite dilution (Table 2). The *D* values determined from MD simulation were generally within statistical variation of the experimental values and reproduced the concentration dependence for the heptasaccharide, although not for the monosaccharide. Notably, the theoretical *D* values at 50 mM remained constant over a relatively large range of system sizes (approximately 10,000 to 40,000 water molecules), regardless of the periodic box size correction proposed by Yeh et al.^43^ (Figure S4). For the largest saccharide, the difference in the *R*_H_ values obtained by NMR at infinite dilution (8.53 Å) versus 50 mM (10.28 Å) equated to a 21% increase in the apparent *R*_H_ at the higher concentration, suggestive of a degree of solute aggregation. The simulations also detected a similar concentration dependence, although the experimental *R*_H_ values for the large heptasaccharide were overestimated by up to 50%.

**Table 2:** Diffusion Coefficients (*D*)­^a^ and Hydrodynamic Radii (*R*_H_)­^b^ for Glc and Maltoheptaose as a Function of Concentration

	Glc		maltoheptaose	
	*D*, infinite dilution	*D*, 50 mM	*D*, infinite dilution	*D*, 50 mM
NMR [DOSY]	0.66 ± 0.01	0.62 ± 0.01	0.29 ± 0.003	0.24 ± 0.003
TIP5P	0.70 ± 0.02	0.70 ± 0.03	0.28 ± 0.03	0.20 ± 0.01
OPC	0.70 ± 0.07	0.76 ± 0.04	0.27 ± 0.03	0.19 ± 0.01

	Glc		maltoheptaose	
	*R*_H_, infinite dilution	*R*_H_, 50 mM	*R*_H_, infinite dilution	*R*_H_, 50 mM
NMR [DOSY]^c^	3.73 ± 0.04	3.95 ± 0.08	8.53 ± 0.10	10.28 ± 0.14
TIP5P^d^	4.48 ± 0.15	4.48 ± 0.23	11.01 ± 1.01	15.55 ± 0.68
OPC^e^	3.97 ± 0.37	3.65 ± 0.23	10.44 ± 0.93	15.28 ± 0.98

a 10^–9^ m^2^/s, standard deviations from 3 replicates.

b In Å, from eq 1.

c From eq 1 with η = 0.00089 kg/m·s (pure water), and temperature = 298 K.

d η (TIP5P) = 0.0007 kg/m·s, and temperature = 298 K.^23^

e η = 0.00079 kg/m·s, and temperature = 298 K.^44^

### Solute Aggregation

To quantify the level of aggregate formation in MD trajectories, the trajectories of Glc, Maltohexaose, and Maltoheptaose were examined for the presence of any carbohydrate-carbohydrate contacts that persisted across at least two MD frames (10 ps/frame). A contact was defined as occurring when any atom in two or more solutes was within 2.85 Å of another monomer in the MD frame. This distance cutoff was selected based on the minimum distance between methanol oxygen atoms in liquid methanol.^45^ An analysis of the MD simulations of 50 mM Glc indicated a very low level of aggregation (Table 3) as expected, given the lack of a significant concentration dependence on its diffusion properties. Nevertheless, the concentration of aggregates was higher in TIP3P water than in the other models, consistent with exaggerated aggregation behavior reported previously for Glc with the GLYCAM06 force field at high concentrations in TIP3P water.^46^ When present, the average aggregate was observed to comprise 2 solute molecules (*S* ≈ 2.0) with a contact lifetime (**τ**) of approximately 0.065 ns, regardless of the carbohydrate or solvent model (Figure 7 and Tables S2–S4).

**Figure 7:**
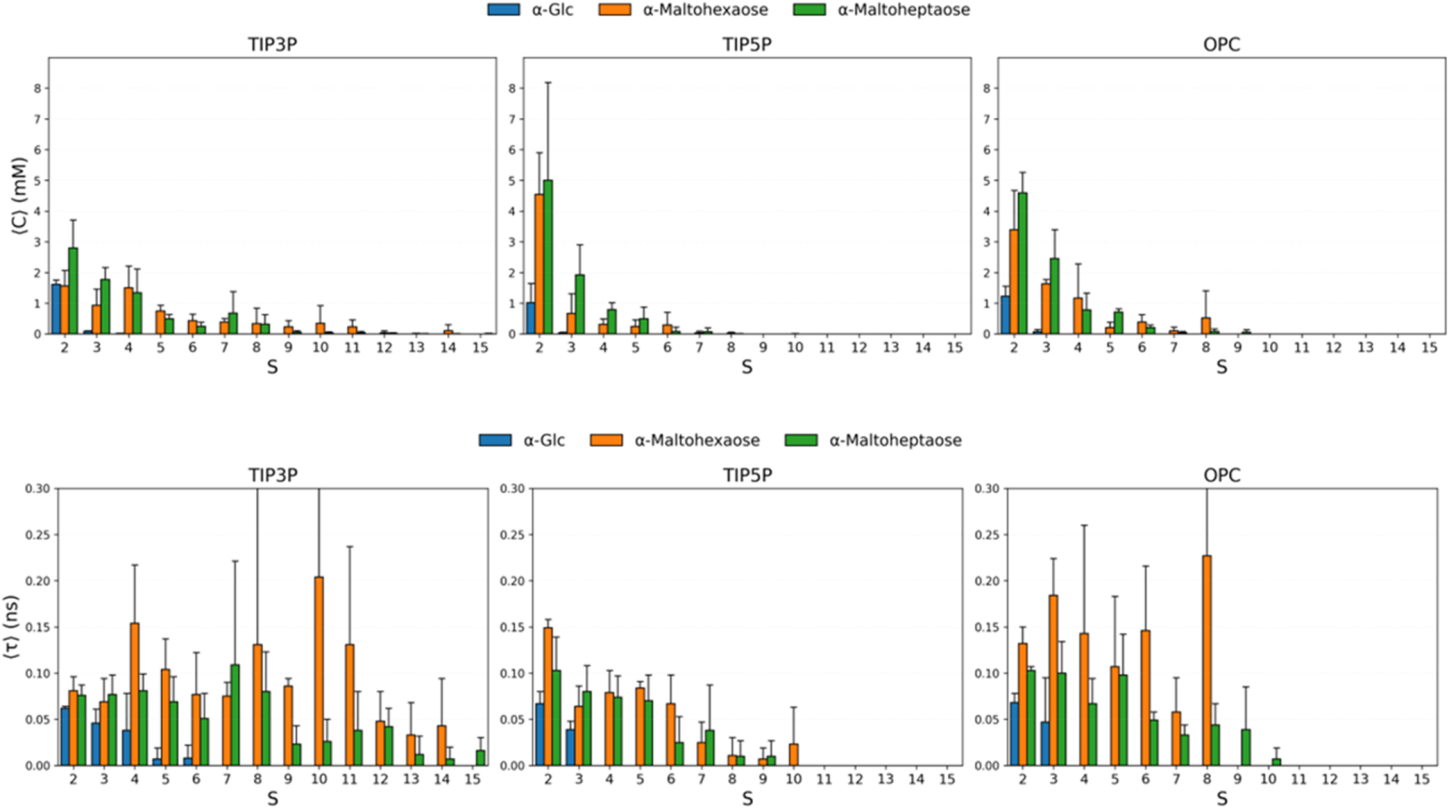
Concentration of aggregates (upper row) and aggregate lifetimes (lower row) as a function of aggregate size in different water models, from Tables S2–S4.

**Table 3:** Free Monomer Concentrations (mM) with the TIP3P, TIP5P, and OPC Water Models

monomeric solute	TIP3P	TIP5P	OPC
α-Glc	46.4 ± 0.34	47.8 ± 1.26	47.3 ± 0.75
α-maltohexaose	16.2 ± 1.66	34.2 ± 2.47	25.4 ± 1.05
α-maltoheptaose	20.6 ± 4.32	27.6 ± 7.79	24.1 ± 2.19

In the case of the larger oligosaccharides, several differences in aggregation behavior were observed between the water models. In TIP3P, aggregates as large as 15-mers were detected, while in TIP5P and OPC, the maximum aggregate sizes were approximately 8- and 9-mers, respectively. Nevertheless, in all water models, a dimer was the predominant aggregate, present at concentrations of 1.6, 4.5, and 3.4 mM (for the hexasaccharide) in TIP3P, TIP5P, and OPC, respectively, and at 2.8, 5.0, and 4.6 mM for the heptasaccharide. Taking into account all aggregates in each system, the concentration of free Glc was reduced from the 50 mM nominal concentration to 46–47 mM regardless of the water model (Table 3).

Based on the data in Table 3, it was evident that the TIP3P water model promoted aggregation significantly more for larger carbohydrates than did either TIP5P or OPC. Interestingly, the lifetimes of the aggregates also depended on the water model; in TIP3P and OPC, aggregates of the hexasaccharide displayed 2- to 3-fold longer lifetimes (0.20 – 0.23 ns) than seen for the heptasaccharide (0.07 – 0.10 ns). In contrast, in TIP5P, there was no difference in the lifetimes of the hexa- and heptasaccharides. Further, in TIP5P, the lifetimes of the aggregates decreased with aggregate size; a feature less evident in the TIP3P or the OPC data. Taken together, the data from the present analysis confirmed that TIP3P promoted the formation of aggregates, particularly for larger solutes, far more so than did TIP5P or OPC. Moreover, the theoretical analysis suggested that aggregation was likely responsible for the experimentally observed concentration-dependent decrease in *D* for the larger carbohydrates (Table 2).

### Hydration Numbers (*n*_H_) and Entrained Waters (*N*_W_)

While the analysis of the RDF data provided values for the number of waters in the first solvation shell (*N*_1_), we sought an approach for estimating the number of tightly entrained waters (*N*_W_), which, in principle, should correspond to the solute hydration number (*n*_H_). Experimental values for *n*_H_ can be inferred from macroscopic solution methods and represent a count of the waters that are tightly bound to a solute.^47,48^ We hypothesized that, if *R*_H_ represented the radius of a sphere with volume $V_{R_{H}}$ (the hydrodynamic volume,^49^) comprising the solute and entrained waters, and *R*_g_ represented the corresponding radius for the unsolvated solute with volume $V_{R_{g}}$ , the difference in these volumes (Δ*V*) should equate to the number of tightly entrained solvent waters (eq 5).

5 $$N_{W}=\frac{V_{R_{H}}-V_{R_{g}}}{V_{W}}=\frac{\Delta V}{V_{W}}$$

where *V*_W_ is the volume (30 Å^3^) of a single water molecule.^50^ Remarkably, the number of tightly entrained waters (*N*_W_) thus derived displayed a near 1:1 correlation with the experimentally determined hydration numbers (*n*_H_) for ten saccharides with reported hydration data Figure S9 and Table 4. This agreement supported the simple spherical-envelope model used to derive eq 5, at least for the selected oligosaccharides. Furthermore, the correlation between *N*_W_ and *n*_H_ suggested that *N*_W_ could be used to predict *n*_H_ when the latter was experimentally unavailable. Given that the entrained waters were presumably stabilized by hydrogen bonds to the polar oxygen atoms in the carbohydrate, the ratio of *N*_W_ (or *n*_H_) to the number of polar atoms (*N*_PA_) in the carbohydrate provided an estimate of approximately 1.1–1.4 water molecules per oxygen atom (Table 4). Knowledge of the ratio of waters per polar atom could then be employed to develop an empirical relationship to derive diffusion properties directly from *R*_g_ values. This approximation would likely be insufficient for polysaccharides whose average shapes were far from spherical, but might still prove convenient for smaller carbohydrates. In proteins, for example, *R*_g_ and *R*_H_ have been shown to exhibit a ratio of approximately 0.77,^51^ if the protein is globular, enabling *D* to be estimated from *R*_g_ for such proteins.

**Table 4:** Theoretical (*N*_W_) and Experimental (*n*_H_) Carbohydrate Hydration Numbers

carbohydrate	*n*_H_ experimental	*N*_W_ *MD*	*N*_PA_ ^a^	*n*_H_/*N*_PA_	*N*_W_/*N*_PA_
Xyl	5.7 ^47,52^	4	5	1.14	0.80
Glc	7.41 ^47,52,53^	5.9	6	1.24	0.98
Gal	9.4 ^52^	4.9	6	1.57	0.82
trehalose	15.3 ^52^	11	11	1.39	1.00
sucrose	10.9 ^47,52^	9.4	11	0.99	0.85
lactose	15.3 ^52^	12	10	1.53	1.20
maltose	14.5 ^52^	13.9	10	1.45	1.39
cellobiose	14.8 ^52^	13.2	10	1.48	1.32
maltotriose	21.3 ^53^	16.7	16	1.33	1.04
maltohexaose	47.4 ^53^	45.6	31	1.53	1.47
average waters per polar atom				**1.36**	**1.09**

a Oxygen atoms were counted as the polar atoms in these molecules.

### Empirical Prediction of Diffusion Properties from *R*_g_

Obtaining accurate diffusion properties by MD simulation requires long replicate simulations to achieve convergence; in contrast, *R*_g_ values converge rapidly, and thus a relationship between *R*_H_ (or *D*) and *R*_g_ would offer a convenient method of estimating carbohydrate transport properties from relatively short MD simulations. Not surprisingly, the *R*_g_ values from MD simulation (Table 5) were directly proportional to the molecular weight (size) of the oligosaccharide, for example, *R*_g_ (Xyl) < *R*_g_ (Fuc) < *R*_g_ (Glc), while *R*_g_ (Glc) = *R*_g_ (Gal), and *R*_g_ (GlcNAc) = *R*_g_ (GalNAc), etc. The fact that the *R*_g_ values from TIP5P and OPC were indistinguishable, despite the observation that each water model led to different distributions of solute aggregates, suggested that the average shape of the monomer (*R*_g_) was unaffected by aggregation. The hydrodynamic radii also displayed a dependence on solute size and showed more sensitivity to the water model. Although the *R*_H_ values from OPC appeared to be slightly more contracted than from TIP5P and were in better agreement with experiment, the differences between the OPC and TIP5P results were not statistically significant (*P* > 0.05, *n* = 3). Overall, the theoretical *R*_H_ values from simulations with TIP5P and OPC water agreed with experimental values within 0.9–1.4 Å. Interestingly, both TIP5P and OPC overestimated the experimental *R*_H_ values, with the deviation from experiment increasing proportionally with oligosaccharide size, up to a maximum of approximately 50%.

**Table 5:** Experimental and Theoretical Hydrodynamic Radii (*R*_H_)­^a^ and Radii of Gyration (*R*_g_)­^a^

	DOSY NMR^b^	MD TIP5P^c^	MD OPC^d^	MD TIP5P	MD OPC
	*R*_H_	*R*_H_	*R*_H_	*R*_g_	*R*_g_
Xyl	3.46 ± 0.05	3.97 ± 0.34	3.52 ± 0.23	2.34 ± 0.03	2.34 ± 0.04
Fuc	3.64 ± 0.16	4.10 ± 0.33	3.89 ± 0.16	2.54 ± 0.02	2.53 ± 0.02
Glc	3.93 ± 0.06	4.39 ± 0.20	3.74 ± 0.25	2.64 ± 0.04	2.64 ± 0.04
Gal	3.77 ± 0.12	4.36 ± 0.42	3.81 ± 0.32	2.63 ± 0.03	2.62 ± 0.04
GlcA	4.02 ± 0.07	4.59 ± 0.25	4.16 ± 0.31	2.55 ± 0.02	2.55 ± 0.02
GlcNAc	4.30 ± 0.08	4.69 ± 0.18	4.46 ± 0.22	3.24 ± 0.03	3.23 ± 0.04
GalNAc	4.30 ± 0.08	4.76 ± 0.20	4.42 ± 0.21	3.23 ± 0.03	3.22 ± 0.03
Neu5Ac	5.22 ± 0.11	5.38 ± 0.29	5.58 ± 0.34	3.48 ± 0.04	3.47 ± 0.04
α,α-trehalose	5.11 ± 0.11	5.89 ± 0.33	5.88 ± 0.50	3.79 ± 0.05	3.80 ± 0.06
sucrose	4.81 ± 0.19	6.12 ± 0.36	5.42 ± 0.43	3.53 ± 0.06	3.53 ± 0.06
lactose	5.22 ± 0.45	6.57 ± 0.56	5.94 ± 0.39	3.84 ± 0.05	3.82 ± 0.05
maltose	5.33 ± 0.12	6.43 ± 0.37	6.01 ± 0.53	3.73 ± 0.05	3.73 ± 0.05
cellobiose	5.33 ± 0.23	6.50 ± 0.69	5.47 ± 0.44	3.85 ± 0.07	3.85 ± 0.05
tetra–O-Ac-Glc-α-OMe	4.63 ± 0.17	6.00 ± 0.44	6.01 ± 0.80	4.08 ± 0.06	4.08 ± 0.07
maltotriose	6.13 ± 0.62	8.21 ± 0.65	7.47 ± 0.61	4.81 ± 0.09	4.82 ± 0.08
cellotriose	6.54 ± 0.35	8.32 ± 0.22	7.90 ± 0.68	5.22 ± 0.07	5.20 ± 0.08
maltohexaose	9.26 ± 0.17	14.51 ± 0.68	13.16 ± 1.27	7.76 ± 0.27	7.76 ± 0.29
maltoheptaose	10.22 ± 0.11	15.60 ± 0.78	14.94 ± 0.81	8.70 ± 0.38	8.69 ± 0.38

a Å.

b From eq 1 with η = 0.00089 kg/m·s (pure water), and temperature = 298 K.

c η (TIP5P) = 0.0007 kg/m·s, and temperature = 298 K.^23^

d η = 0.00079 kg/m·s, and temperature = 298 K.^44^

By rearranging eq 5, *R*_H_ could potentially be estimated from *R*_g_ augmented with an increase in solute radius arising from the entrained water molecules (eq 6):

6 $$R_{H}=\sqrt[{3}]{\frac{3}{4\pi }\left(NV_{W}+V_{R_{g}}\right)}=\sqrt[{3}]{\frac{3V_{w}}{4\pi }N+{R_{g}}^{3}}$$

where *N* = the number of entrained waters either from *n*_H_ or *N*_W_.

A comparison of experimentally measured *D* values for 18 carbohydrates versus those measured by MD simulation and those derived empirically by employing the *R*_H_ values from eq 6 is presented in Table 6. Remarkably, the empirical model was found to be at least as accurate as the MD simulations in reproducing the experimental values, suggesting that the use of *R*_g_ from MD augmented by a simple polar-group heuristic could provide a fast, reasonable estimate of the diffusion properties. Such a high-throughput empirical approach could be very convenient for the virtual design of oligosaccharides with targeted diffusion properties. However, for high-*M*_W_ carbohydrates, where ${R_{g}}^{3}>>\frac{3V_{w}}{4\pi }N$ , eq 6 reduces to *R*_H_ ≈ *R*_g_, which would make the sphere-envelope model inappropriate for large polysaccharides. In fact, in large extended polysaccharides, such as hyaluronic acid^49^ and dextran,^54^ *R*_g_ may be larger than *R*_H_ due to the fact that *R*_g_ increases with the mean-square distance of polymer chain segments from the center of mass, whereas *R*_H_ depends on translational diffusion and scales more weakly with polymer length.^51,55^

**Table 6:** Diffusion Coefficients (*D*)­^a^ Measured by DOSY NMR, MD Simulation, and Empirically

	DOSY NMR	MD TIP5P	MD OPC	empirical (*R*_H_ from eq 6)^b^
Xyl	0.71 ± 0.01	0.79 ± 0.07	0.79 ± 0.05	0.66
Fuc	0.68 ± 0.03	0.76 ± 0.06	0.71 ± 0.03	0.64
Glc	0.63 ± 0.01	0.71 ± 0.03	0.74 ± 0.05	0.61
Gal	0.65 ± 0.02	0.72 ± 0.07	0.73 ± 0.06	0.61
GlcA^c^	0.61 ± 0.01	0.68 ± 0.04	0.67 ± 0.05	0.59
GlcNAc^d^	0.57 ± 0.01	0.67 ± 0.02	0.62 ± 0.03	0.55
GalNAc^d^	0.57 ± 0.01	0.66 ± 0.03	0.63 ± 0.03	0.55
Neu5Ac^c^,^d^	0.47 ± 0.01	0.58 ± 0.03	0.50 ± 0.03	0.50
α,α-trehalose	0.48 ± 0.01	0.53 ± 0.03	0.47 ± 0.04	0.47
sucrose	0.51 ± 0.02	0.51 ± 0.03	0.51 ± 0.04	0.48
lactose	0.47 ± 0.04	0.48 ± 0.04	0.47 ± 0.03	0.48
maltose	0.46 ± 0.01	0.49 ± 0.03	0.46 ± 0.04	0.48
cellobiose	0.46 ± 0.02	0.48 ± 0.05	0.51 ± 0.04	0.48
tetra–O-Ac-Glc-α-OMe^e^	0.53 ± 0.02	0.52 ± 0.04	0.46 ± 0.06	0.47
maltotriose	0.40 ± 0.04	0.38 ± 0.03	0.37 ± 0.03	0.41
cellotriose	0.38 ± 0.02	0.38 ± 0.01	0.35 ± 0.03	0.39
maltohexaose	0.27 ± 0.01	0.22 ± 0.01	0.21 ± 0.02	0.27
maltoheptaose	0.24 ± 0.003	0.20 ± 0.01	0.19 ± 0.01	0.25
**MAE** ^f^		**0.05 ± 0.04**	**0.04 ± 0.03**	**0.02 ± 0.02**

a 10^–9^ m^2^/s at 50 mM; values averaged over both anomers.

b Computed with *N* = 1.1 *N*_PA_.

c Assuming a carboxylate group contributes two polar atoms.

d Assuming an amide group contributes two polar atoms.

e Assuming an ester group contributes two polar atoms.

f Mean absolute error.

## Conclusions

This study demonstrated the capability and limitations of MD simulations with the GLYCAM06 force field to provide a physically grounded framework for understanding carbohydrate diffusion in aqueous environments. In the low-*M*_W_ regime, GLYCAM06 was found to reproduce experimental diffusion coefficients with high accuracy when it was paired with an appropriate water model. The choice of water model was especially critical; OPC and TIP5P were markedly more accurate than the simpler TIP3P model. An analysis of solvation properties revealed substantial variation in solvation shell structure, but despite this, the total number of water molecules associated with each carbohydrate and the average radial distance of the first hydration shell were similar, suggesting that diffusion is likely more sensitive to bulk water properties, such as viscosity than to local solute–solvent (frictional) interactions.

As the carbohydrate size increased, an increased tendency to form aggregates was observed. This aggregation significantly retarded diffusion, consistent with experimentally observed concentration effects. The MD simulations captured this effect, although with a systematic tendency to overestimate its magnitude. Even with this limitation, MD simulation represents a valuable alternative for predicting diffusion coefficients of carbohydrates compared for example to empirical power-law relationships, whose performance is finally tuned to a specific chemical class and therefore should not be generalized to different chemistries.^56^ Simulations instead provide a transferable mechanistic description of how specific features, such as hydrogen bonding, solvation shell structure, and intermolecular association, govern translational mobility.

A key outcome of this work was the development of a hybrid approach to the determination of the number of entrained waters (*N*_W_), which provides a practical alternative to the experimental hydration number (*n*_H_) measurements. Further, by examining the relationship between *N*_W_ and the number of polar groups, a simple relationship was revealed in which each polar group was associated with approximately one tightly bound water, providing a chemically intuitive rule to estimate *N*_W_ for carbohydrates. This observation could be further explored either by MD simulation or empirically to fine-tune this relationship for varying polar atom classes.

Building on this result, a eq 6 was derived to predict *R*_H_ and *D* for carbohydrates using readily accessible theoretical *R*_g_ values coupled with the empirical estimate of *N*_W_. This equation reproduced experimental *R*_H_ values with a near 1:1 correlation (Figure S10), demonstrating that *R*_H_ and *D* for mono- and oligosaccharides could be predicted accurately without direct diffusion measurements.

Together, these findings highlight the dual utility of MD simulations: they not only can reproduce known experimental trends but also provide new theoretical constructs that expand the interpretive toolkit for carbohydrate biophysics. By establishing a consistent relationship among chemical functionality, hydration, and diffusion, this work opens the door to more predictive modeling of carbohydrate properties across size and compositional scales. The results presented here are expected to be broadly applicable to glycomaterial design and offer a framework for future efforts to connect simulation, experiment, and theory in the study of condensed soft matter.

## Supplementary Material



## Data Availability

All simulation input files and analysis scripts used in this work are available on GitHub (https://github.com/holmess2013/Diffusion_Simulations_and_Analysis).

## Notes

System density, temperature, and total energy versus time for β-d-Glc from proof of concept NVE simulations, system size effects on the diffusion of β-D-Glc, ^1^H NMR spectrum of tetra–O-Ac-Glc-α-OMe, diffusion coefficients of both anomers for all reducing sugars, water occupancy maps at different occupancy cutoffs for β-d-Xyl, infinite dilution diffusion coefficient estimation for β-d-Glc and α-maltoheptaose, correlation of *N*_W_ versus *n*_H_ for ten sugars from the data set, correlation of empirical *R*_H_ versus experimental *R*_H_, and tabulated values of measured and predicted diffusion coefficients, aggregation characteristics, *N*_W_, *N*_W_/polar group, and *n*_H_ (PDF)
