# Supplementary material for: Exploring Diffusion and Aggregation Behaviors in Carbohydrate Solutions
Source: J Phys Chem B. 2026 Jan 8;130(3):1106–16. doi: 10.1021/acs.jpcb.5c07628 (PMC12833843; doi:10.1021/acs.jpcb.5c07628)
Supplement: Supplementary file 1 [file jp5c07628_si_001.pdf]

Exploring Diffusion and Aggregation Behaviors in Carbohydrate Solutions

Samuel G. Holmes, Sawsan Mahmoud, and Robert J. Woods*

Complex Carbohydrate Research Center, University of Georgia, Athens, GA 30602

#	Contents	Pg.
**Figure S1**	Density vs time of β-D-Glc simulation	S2
**Figure S2**	Temperature vs time of β-D-Glc	S3
**Figure S3**	Total energy vs time of β-D-Glc	S4
**Figure S4**	Study of system size effects on the diffusion coefficient of β-D-Glc	S5
**Figure S5**	HNMR spectrum of Tetra-O-Ac-Glc-α-OMe	S6
**Figure S6**	Diffusion coefficients for both anomers of reducing sugars	S7
**Figure S7**	Water occupancy maps for β-D-Xyl at different occupancy cutoffs	S8
**Figure S8**	Infinite dilution diffusion coefficient estimation for β-D-Glc and α-Maltoheptaose	S9
**Figure S9**	Correlation of N_W versus n_H for ten carbohydrates	S10
**Figure S10**	Correlation of empirical R_H versus experimental R_H	S11
**Table S1**	Measured and predicted diffusion coefficients	S12
**Table S2**	Aggregation characteristics from MD simulations of carbohydrates at 50 mM in the TIP3P water model	S13
**Table S3**	Aggregation characteristics from MD simulations of carbohydrates at 50 mM in the TIP5P water model	S14
**Table S4**	Aggregation characteristics from MD simulations of carbohydrates at 50 mM in the OPC water model	S15
**Table S5**	N_W, N_W/polar group and n_H for all carbohydrates	S16

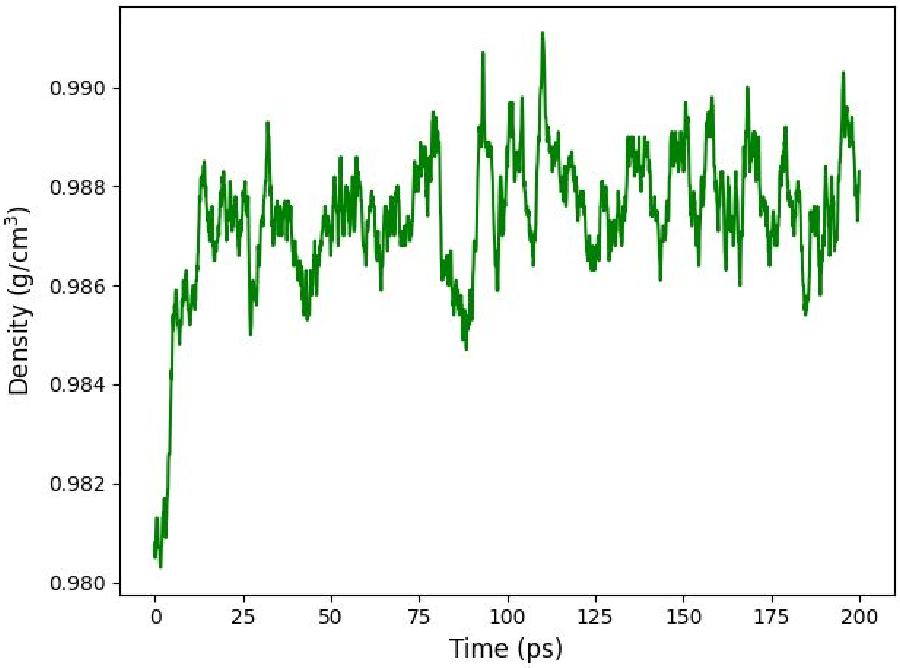

Figure S1. System density vs. time during step 10 of the MD simulation protocol in the NPT ensemble from a β-D-Glc simulation with the TIP5P water model.

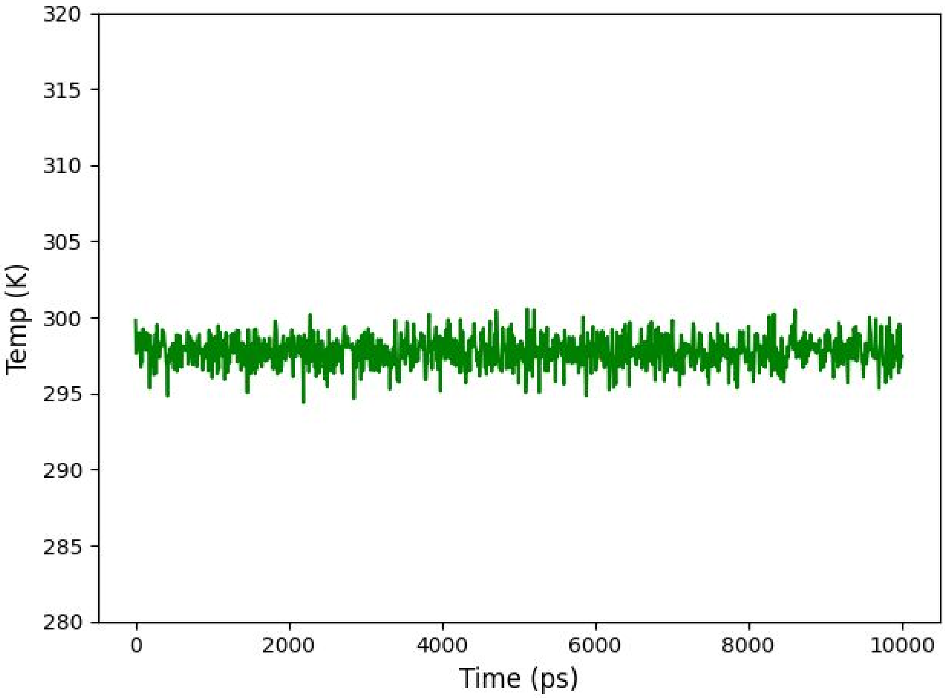

Figure S2: Temperature vs. time during the NVE production phase from a β-D-Glc simulation with the TIP5P water model.

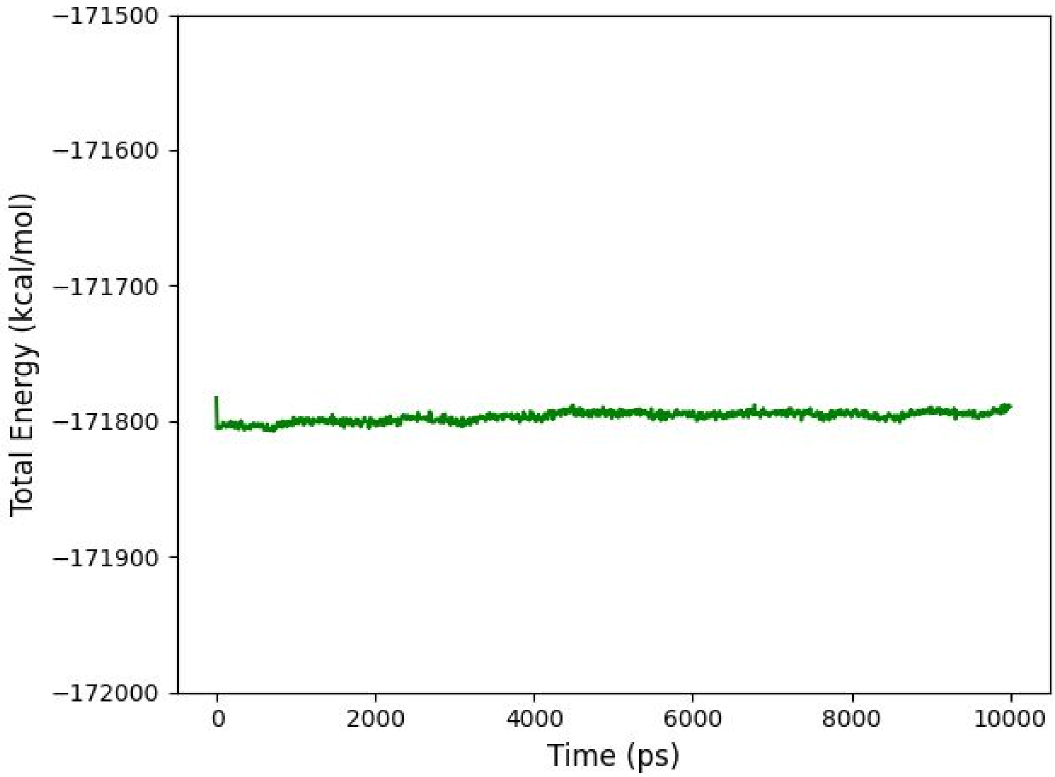

Figure S3. Total energy vs. time during the NVE production phase from a β-D-Glc simulation with the TIP5P water model.

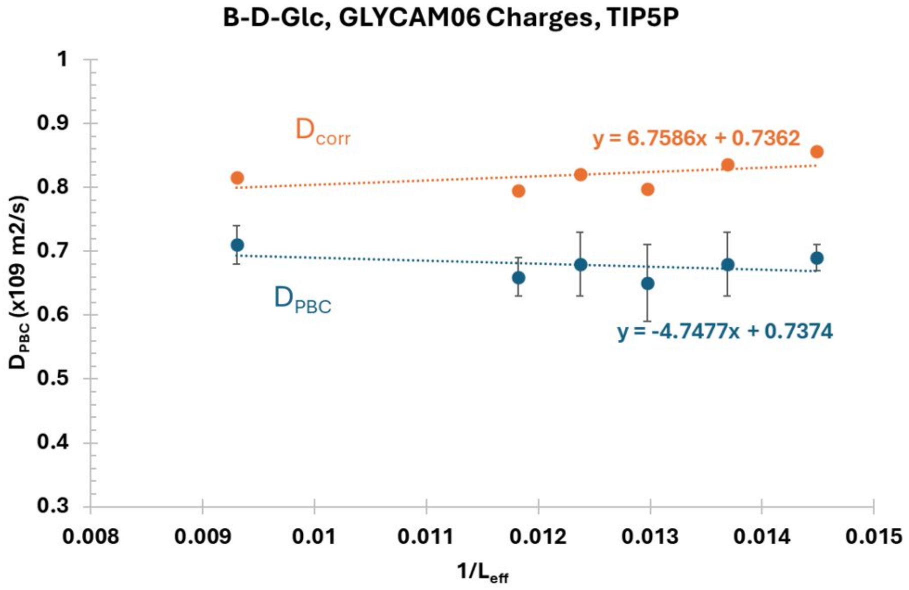

Figure S4. System-size effects on the predicted diffusion coefficient of β-D-Glc using the TIP5P water model. For each data point, the system size ranged from 10,000 – 40,000 molecules, where each simulation had varying amounts of water and a constant number of sugars (20). Additionally, the infinite cubic box size correction as reported previously [1] was applied to each D_{PBC}. Differences in D_{PBC} and D_{corr} are likely due to several reasons: an octahedral simulation cell rather than cubic, the reported viscosity of TIP5P was used rather than calculating it explicitly, and the simulation conditions are different than those for which the correction was derived.

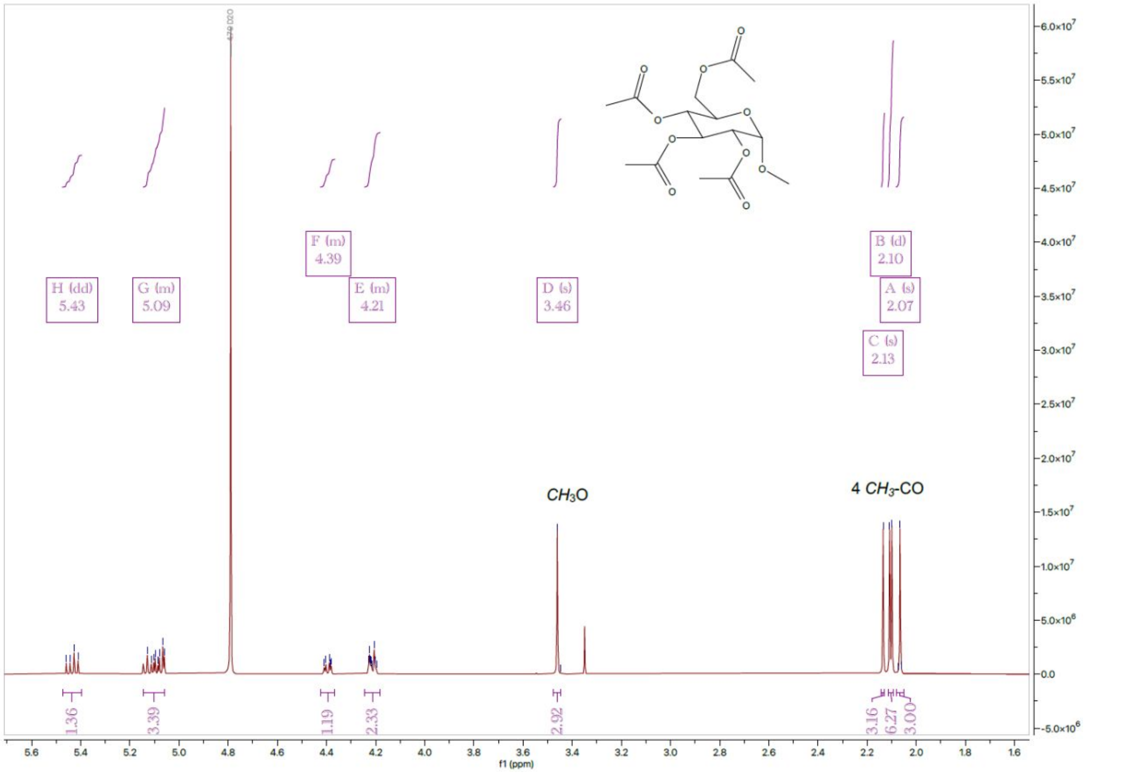

Figure S5. HNMR spectrum of Tetra-O-Ac-Glc-α-OMe in D_2O.

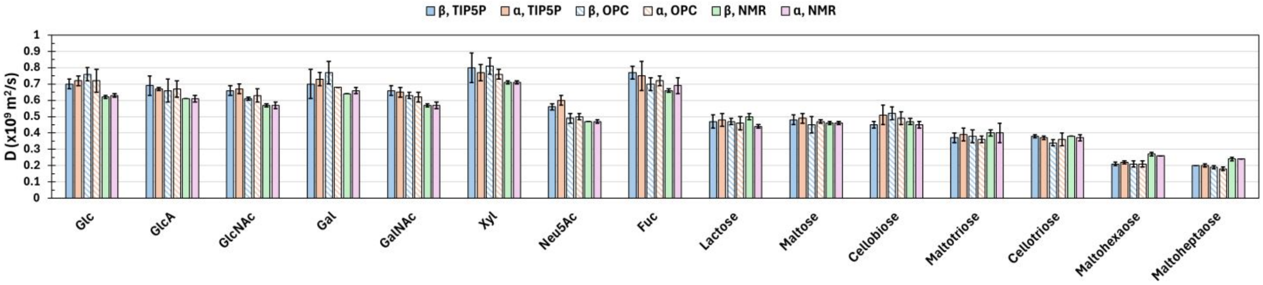

Figure S6. Benchmarking GLYCAM06 predictions of carbohydrate diffusion coefficients with the TIP5P or OPC water models against measured values from DOSY NMR.

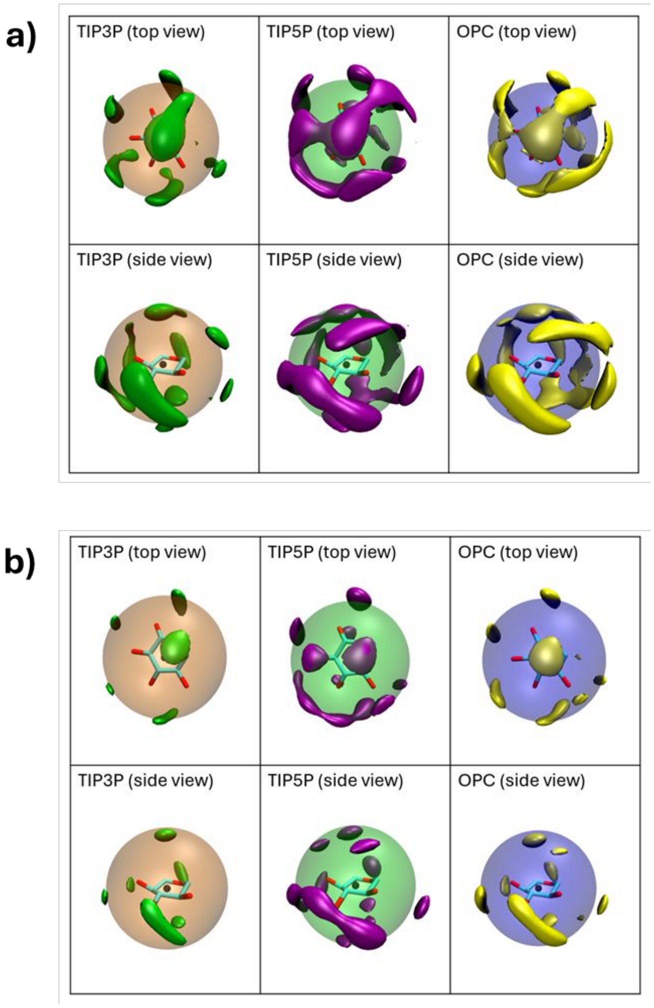

Figure S7. Water occupancy maps of the first solvation shell with occupancy cutoffs of 3.8% (a) and 4.0% (b). The radius of the large sphere (blue, orange, green) corresponds to the maximum peak height in the pertinent RDF curves (Figure 4) and provides an indication of the location of the first solvation shell. The geometric center of the monosaccharide is indicated with a small black sphere. Occupancy maps are displayed from the top of the monosaccharide (upper row of panels) and from the side (lower row).

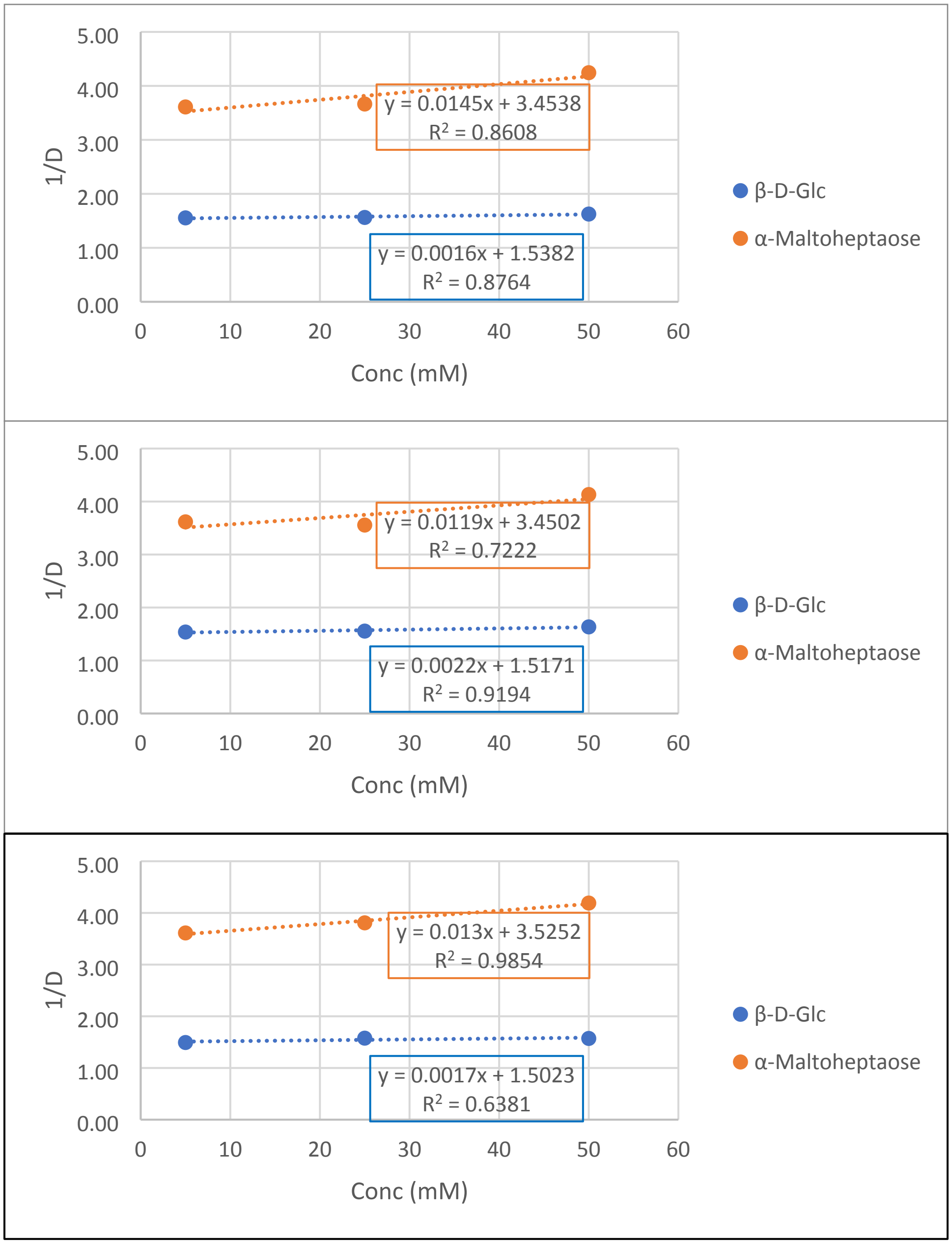

Figure S8. Infinite dilution diffusion coefficient estimations for β-D-Glc and α-Maltoheptaose across three replicate DOSY experiments.

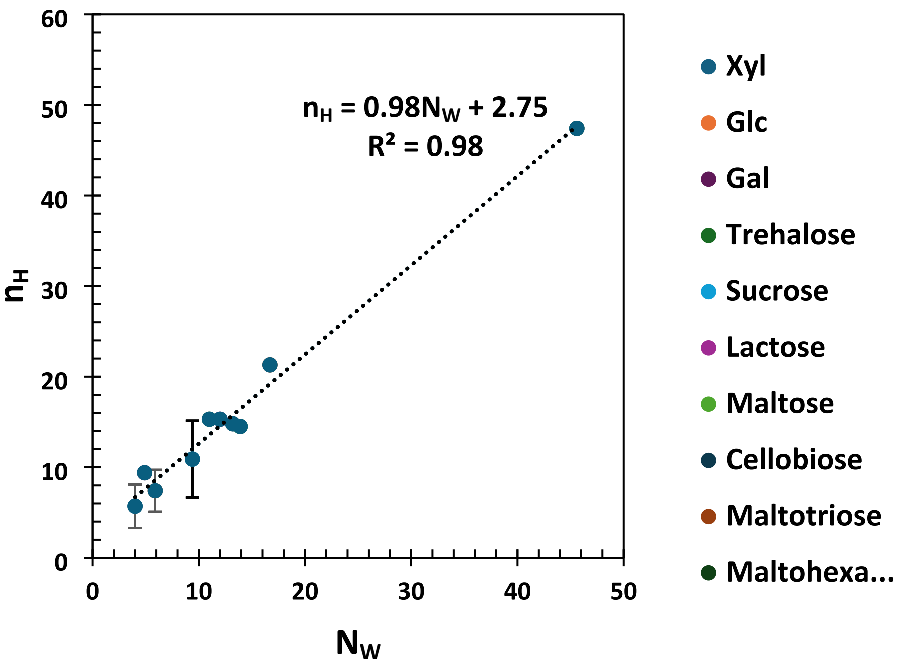

Figure S9. Correlation of N_W versus n_H for ten carbohydrates.

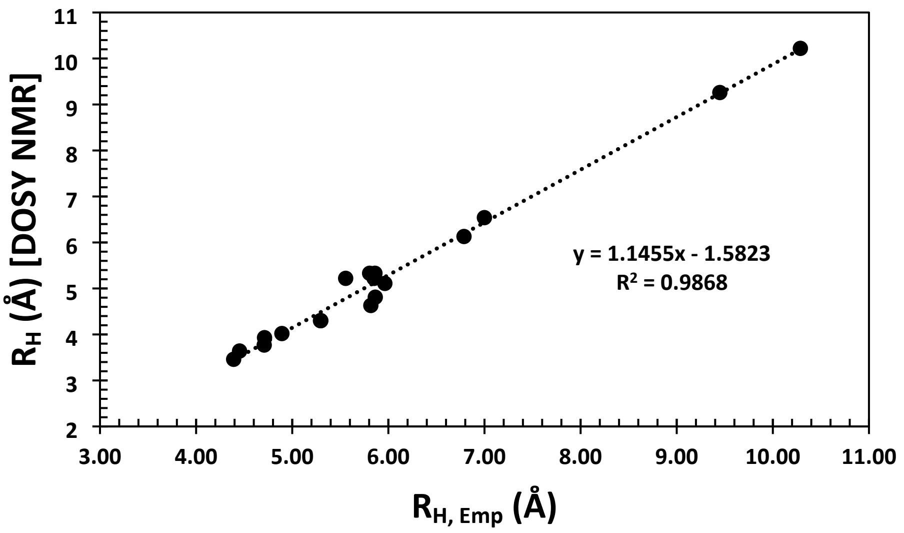

Figure S10. Correlation of empirically estimated R_H versus experimental R_H for ten carbohydrates.

Table S1. Measured and predicted diffusion coefficients (*D*)[a]

	Carbohydrate	M_W (g/Mol)	DOSY NMR[b]	Literature	MD (TIP5P)	MD (OPC)
1	Xyl	150.13	0.71 ± 0.01	0.75 [2], 0.77 [3]	0.79 ± 0.07	0.79 ± 0.05
2	Fuc	164.16	0.68 ± 0.03	-	0.76 ± 0.06	0.71 ± 0.03
3	Glc	180.16	0.63 ± 0.01	0.63 [4], 0.67 [5], 0.68 [3], 0.68 [6]	0.71 ± 0.03	0.74 ± 0.05
4	Gal	180.16	0.65 ± 0.02	0.71 [3]	0.72 ± 0.07	0.73 ± 0.06
5	GlcA	194.14	0.61 ± 0.01	-	0.68 ± 0.04	0.67 ± 0.05
6	GlcNAc	221.21	0.57 ± 0.01	0.62 [7]	0.67 ± 0.02	0.62 ± 0.03
7	GalNAc	221.21	0.57 ± 0.01	-	0.66 ± 0.03	0.63 ± 0.03
8	Neu5Ac	309.27	0.47 ± 0.01	-	0.58 ± 0.03	0.50 ± 0.03
9	α,α-Trehalose	342.3	0.48 ± 0.01	0.61 [8],	0.53 ± 0.03	0.47 ± 0.04
10	Sucrose	342.3	0.51 ± 0.02	0.52 [9], 0.52 [6], 0.52 [5], 0.53 [10], 0.55 [3]	0.51 ± 0.03	0.51 ± 0.04
11	Lactose	342.3	0.47 ± 0.04	0.57 [10]	0.48 ± 0.04	0.47 ± 0.03
12	Maltose	342.3	0.46 ± 0.01	-	0.49 ± 0.03	0.46 ± 0.04
13	Cellobiose	342.3	0.46 ± 0.02	0.52 [11]	0.48 ± 0.05	0.51 ± 0.04
14	Tetra-O-Ac-Glc-α-OMe	362.12	0.53 ± 0.02	-	0.52 ± 0.04	0.46 ± 0.06
15	Maltotriose	504.44	0.40 ± 0.04	-	0.38 ± 0.03	0.37 ± 0.03
16	Cellotriose	504.44	0.38 ± 0.02	0.42 [11]	0.38 ± 0.01	0.35 ± 0.03
17	Maltohexaose	990.86	0.27 ± 0.00	-	0.22 ± 0.01	0.21 ± 0.02
18	Maltoheptaose	1153.0	0.24 ± 0.00	-	0.2 ± 0.01	0.19 ± 0.01

[a]10^9 m^2/s

Table S2. Aggregation[a] characteristics from MD simulations of carbohydrates at 50 mM in the TIP3P water model.

	Glc		Maltohexaose		Maltoheptaose	
**S**	< C >[b] mM	< **τ**>[c] ns	< C > mM	< **τ**> ns	< C > mM	< **τ**> ns
**1**	46.41 ± 0.34	-	16.23 ± 1.66	-	20.55 ± 4.32	-
**2**	1.61 ± 0.14	0.06 ± 0.00	1.56 ± 0.51	0.08 ± 0.01	2.80 ± 0.91	0.08 ± 0.01
**3**	0.09 ± 0.01	0.05 ± 0.01	0.93 ± 0.53	0.07 ± 0.03	1.77 ± 0.39	0.08 ± 0.02
**4**	0.01 ± 0.01	0.04 ± 0.04	1.50 ± 0.71	0.15 ± 0.06	1.34 ± 0.77	0.08 ± 0.02
**5**	0.00 ± 0.00	0.01 ± 0.01	0.75 ± 0.18	0.10 ± 0.03	0.49 ± 0.14	0.07 ± 0.03
**6**	0.00 ± 0.00	0.01 ± 0.01	0.43 ± 0.21	0.08 ± 0.04	0.25 ± 0.13	0.05 ± 0.03
**7**	0.00 ± 0.00	0.00 ± 0.00	0.39 ± 0.11	0.07 ± 0.01	0.68 ± 0.70	0.11 ± 0.11
**8**	0.00 ± 0.00	0.00 ± 0.00	0.33 ± 0.50	0.13 ± 0.18	0.32 ± 0.30	0.08 ± 0.04
**9**	0.00 ± 0.00	0.00 ± 0.00	0.23 ± 0.20	0.09 ± 0.01	0.05 ± 0.05	0.02 ± 0.02
**10**	0.00 ± 0.00	0.00 ± 0.00	0.34 ± 0.58	0.20 ± 0.33	0.03 ± 0.03	0.03 ± 0.02
**11**	0.00 ± 0.00	0.00 ± 0.00	0.23 ± 0.23	0.13 ± 0.11	0.04 ± 0.04	0.04 ± 0.04
**12**	0.00 ± 0.00	0.00 ± 0.00	0.04 ± 0.06	0.05 ± 0.03	0.02 ± 0.02	0.04 ± 0.02
**13**	0.00 ± 0.00	0.00 ± 0.00	0.01 ± 0.01	0.03 ± 0.04	0.00 ± 0.01	0.01 ± 0.02
**14**	0.00 ± 0.00	0.00 ± 0.00	0.11 ± 0.19	0.04 ± 0.05	0.00 ± 0.01	0.01 ± 0.01
**15**	0.00 ± 0.00	0.00 ± 0.00	0.00 ± 0.00	0.00 ± 0.00	0.01 ± 0.01	0.02 ± 0.01
**16**	0.00 ± 0.00	0.00 ± 0.00	0.00 ± 0.00	0.00 ± 0.00	0.00 ± 0.00	0.01 ± 0.01
**17**	0.00 ± 0.00	0.00 ± 0.00	0.00 ± 0.00	0.00 ± 0.00	0.00 ± 0.01	0.02 ± 0.03
**18**	0.00 ± 0.00	0.00 ± 0.00	0.00 ± 0.00	0.00 ± 0.00	0.00 ± 0.00	0.00 ± 0.00
**19**	0.00 ± 0.00	0.00 ± 0.00	0.00 ± 0.00	0.00 ± 0.00	0.00 ± 0.00	0.00 ± 0.00
**20**	0.00 ± 0.00	0.00 ± 0.00	0.00 ± 0.00	0.00 ± 0.00	0.00 ± 0.00	0.00 ± 0.00

[a]Based on a minimum monosaccharide-monosaccharide distance of 2.85 Å [12]. [b]Aggregate concentration. [c]Aggregate lifetime.

Table S3. Aggregation[a] characteristics from MD simulations of carbohydrates at 50 mM in the TIP5P water model.

	Glc		Maltohexaose		Maltoheptaose	
**S**	< C >[b] mM	< **τ**>[c] ns	< C > mM	< **τ**> ns	< C > mM	< **τ**> ns
**1**	47.83 ± 1.26	-	34.23 ± 2.47	-	27.56 ± 7.79	-
**2**	1.02 ± 0.62	0.07 ± 0.01	4.54 ± 1.36	0.15 ± 0.01	5.00 ± 3.18	0.10 ± 0.04
**3**	0.03 ± 0.02	0.04 ± 0.01	0.67 ± 0.64	0.06 ± 0.02	1.92 ± 0.98	0.08 ± 0.03
**4**	0.00 ± 0.00	0.00 ± 0.00	0.31 ± 0.17	0.08 ± 0.02	0.79 ± 0.23	0.07 ± 0.02
**5**	0.00 ± 0.00	0.00 ± 0.00	0.24 ± 0.22	0.08 ± 0.01	0.49 ± 0.38	0.07 ± 0.03
**6**	0.00 ± 0.00	0.00 ± 0.00	0.29 ± 0.41	0.07 ± 0.03	0.08 ± 0.14	0.03 ± 0.03
**7**	0.00 ± 0.00	0.00 ± 0.00	0.04 ± 0.05	0.03 ± 0.02	0.07 ± 0.12	0.04 ± 0.05
**8**	0.00 ± 0.00	0.00 ± 0.00	0.02 ± 0.03	0.01 ± 0.02	0.00 ± 0.01	0.01 ± 0.02
**9**	0.00 ± 0.00	0.00 ± 0.00	0.00 ± 0.00	0.01 ± 0.01	0.00 ± 0.00	0.01 ± 0.02
**10**	0.00 ± 0.00	0.00 ± 0.00	0.00 ± 0.01	0.02 ± 0.04	0.00 ± 0.00	0.00 ± 0.00
**11**	0.00 ± 0.00	0.00 ± 0.00	0.00 ± 0.00	0.00 ± 0.00	0.00 ± 0.00	0.00 ± 0.00
**12**	0.00 ± 0.00	0.00 ± 0.00	0.00 ± 0.00	0.00 ± 0.00	0.00 ± 0.00	0.00 ± 0.00
**13**	0.00 ± 0.00	0.00 ± 0.00	0.00 ± 0.00	0.00 ± 0.00	0.00 ± 0.00	0.00 ± 0.00
**14**	0.00 ± 0.00	0.00 ± 0.00	0.00 ± 0.00	0.00 ± 0.00	0.00 ± 0.00	0.00 ± 0.00
**15**	0.00 ± 0.00	0.00 ± 0.00	0.00 ± 0.00	0.00 ± 0.00	0.00 ± 0.00	0.00 ± 0.00
**16**	0.00 ± 0.00	0.00 ± 0.00	0.00 ± 0.00	0.00 ± 0.00	0.00 ± 0.00	0.00 ± 0.00
**17**	0.00 ± 0.00	0.00 ± 0.00	0.00 ± 0.00	0.00 ± 0.00	0.00 ± 0.00	0.00 ± 0.00
**18**	0.00 ± 0.00	0.00 ± 0.00	0.00 ± 0.00	0.00 ± 0.00	0.00 ± 0.00	0.00 ± 0.00
**19**	0.00 ± 0.00	0.00 ± 0.00	0.00 ± 0.00	0.00 ± 0.00	0.00 ± 0.00	0.00 ± 0.00
**20**	0.00 ± 0.00	0.00 ± 0.00	0.00 ± 0.00	0.00 ± 0.00	0.00 ± 0.00	0.00 ± 0.00

[a]Based on a minimum monosaccharide-monosaccharide distance of 2.85 Å [12]. [b]Aggregate concentration. [c]Aggregate lifetime.

Table S4. Aggregation[a] characteristics from MD simulations of carbohydrates at 50 mM in the OPC water model

	Glc		Maltohexaose		Maltoheptaose	
**S**	< C >[b] mM	< **τ**>[c] ns	< C > mM	< **τ**> ns	< C > mM	< **τ**> ns
1	47.31 ± 0.75	-	25.42 ± 1.05	-	24.09 ± 2.19	-
2	1.23 ± 0.32	0.07 ± 0.01	3.39 ± 1.28	0.13 ± 0.02	4.59 ± 0.67	0.10 ± 0.00
3	0.07 ± 0.07	0.05 ± 0.05	1.63 ± 0.14	0.18 ± 0.04	2.45 ± 0.94	0.10 ± 0.03
4	0.00 ± 0.00	0.00 ± 0.00	1.17 ± 1.11	0.14 ± 0.12	0.78 ± 0.54	0.07 ± 0.03
5	0.00 ± 0.00	0.00 ± 0.00	0.20 ± 0.18	0.11 ± 0.08	0.71 ± 0.11	0.10 ± 0.04
6	0.00 ± 0.00	0.00 ± 0.00	0.39 ± 0.23	0.15 ± 0.07	0.20 ± 0.09	0.05 ± 0.01
7	0.00 ± 0.00	0.00 ± 0.00	0.10 ± 0.12	0.06 ± 0.04	0.04 ± 0.04	0.03 ± 0.01
8	0.00 ± 0.00	0.00 ± 0.00	0.52 ± 0.88	0.23 ± 0.34	0.08 ± 0.08	0.04 ± 0.02
9	0.00 ± 0.00	0.00 ± 0.00	0.00 ± 0.00	0.00 ± 0.00	0.05 ± 0.08	0.04 ± 0.05
10	0.00 ± 0.00	0.00 ± 0.00	0.00 ± 0.00	0.00 ± 0.00	0.00 ± 0.00	0.01 ± 0.01
11	0.00 ± 0.00	0.00 ± 0.00	0.00 ± 0.00	0.00 ± 0.00	0.00 ± 0.00	0.00 ± 0.00
12	0.00 ± 0.00	0.00 ± 0.00	0.00 ± 0.00	0.00 ± 0.00	0.00 ± 0.00	0.00 ± 0.00
13	0.00 ± 0.00	0.00 ± 0.00	0.00 ± 0.00	0.00 ± 0.00	0.00 ± 0.00	0.00 ± 0.00
14	0.00 ± 0.00	0.00 ± 0.00	0.00 ± 0.00	0.00 ± 0.00	0.00 ± 0.00	0.00 ± 0.00
15	0.00 ± 0.00	0.00 ± 0.00	0.00 ± 0.00	0.00 ± 0.00	0.00 ± 0.00	0.00 ± 0.00
16	0.00 ± 0.00	0.00 ± 0.00	0.00 ± 0.00	0.00 ± 0.00	0.00 ± 0.00	0.00 ± 0.00
17	0.00 ± 0.00	0.00 ± 0.00	0.00 ± 0.00	0.00 ± 0.00	0.00 ± 0.00	0.00 ± 0.00
18	0.00 ± 0.00	0.00 ± 0.00	0.00 ± 0.00	0.00 ± 0.00	0.00 ± 0.00	0.00 ± 0.00
19	0.00 ± 0.00	0.00 ± 0.00	0.00 ± 0.00	0.00 ± 0.00	0.00 ± 0.00	0.00 ± 0.00
20	0.00 ± 0.00	0.00 ± 0.00	0.00 ± 0.00	0.00 ± 0.00	0.00 ± 0.00	0.00 ± 0.00

[a]Based on a minimum monosaccharide-monosaccharide distance of 2.85 Å [12]. [b]Aggregate concentration. [c]Aggregate lifetime.

Table S5. N_W and N_W /polar group to measure tightly bound waters at the molecular and atomic levels, respectively.

Carbohydrate	M_W (g/mol)	Total polar (O,N) groups	V_{R_g} (Å³)	V_{R_H} (Å³)	ΔV (Å³)	N_W	N_W per Polar Group	n_h
Xyl	150.1	5	53.3	173.4	120.1	4.0	0.8	5.7 ± 2.4 [13, 14]
Fuc	164.2	5	68.2	201.9	133.7	4.5	0.9	-
Glc	180.2	6	76.6	254.1	177.5	5.9	1.0	7.41 ± 2.3 [13-15]
Gal	180.2	6	76.2	224.3	148.2	4.9	0.8	9.4 [14]
GlcA	194.1	7	69.0	272.0	203.0	6.8	1.0	-
GlcNAc	221.2	8	141.7	332.9	191.1	6.4	0.8	-
GalNAc	221.2	8	140.4	332.9	192.4	6.4	0.8	-
Neu5Ac	309.3	9	176.4	595.5	419.1	14.0	1.6	-
Trehalose	342.3	11	227.9	558.6	330.7	11.0	1.0	15.3 [14]
Sucrose	342.3	11	184.2	465.9	281.8	9.4	0.9	10.9 ± 4.2 [13, 14]
Lactose	342.3	10	236.1	595.5	359.4	12.0	1.2	15.3 [14]
Maltose	342.3	10	216.4	633.9	417.5	13.9	1.4	14.5 [14]
Cellobiose	342.3	10	238.9	633.9	395.0	13.2	1.3	14.8 [14]
Tetra-O-Ac-Glc-α-OMe	362.1	9	284.3	415.5	131.2	4.4	0.5	-
Maltotriose	504.4	14	464.5	964.4	499.9	16.7	1.2	21.3 [15]
Cellotriose	504.4	14	595.5	1171.1	575.6	19.2	1.4	-
Maltohexaose	990.9	26	1956.4	3324.3	1367.9	45.6	1.8	47.4 [15]
Maltoheptaose	1153.0	30	2756.9	4469.1	1712.2	57.1	1.9	-

References

(1) Yeh, I.-C.; Hummer, G. System-size dependence of diffusion coefficients and viscosities from molecular dynamics simulations with periodic boundary conditions. *J. Phys. Chem. B* **2004**, *108* (40), 15873–15879. DOI: 10.1021/jp0477147.

(2) Ueadaira, H.; Uedaira, H. Diffusion coefficients of xylose and maltose in aqueous solution. *Bull. Chem. Soc. Jpn.* **1969**, *42* (8), 2140–2142.

(3) Mogi, N.; Sugai, E.; Fuse, Y.; Funazukuri, T. Infinite dilution binary diffusion coefficients for six sugars at 0.1 MPa and temperatures from (273.2 to 353.2) K. *J. Chem. Eng. Data* **2007**, *52* (1), 40–43. DOI: 10.1021/je0601816.

(4) Nagy, L.; Gyetvai, G.; Nagy, G. Determination of the diffusion coefficient of monosaccharides with scanning electrochemical microscopy (SECM). *Electroanalysis* **2009**, *21* (3-5), 542–549. DOI: https://doi.org/10.1002/elan.200804442.

(5) Longsworth, L. G. Diffusion measurements, at 25°, of aqueous solutions of amino acids, peptides and sugars. *J. Am. Chem. Soc.* **1953**, *75* (22), 5705–5709. DOI: 10.1021/ja01118a065.

(6) Gladden, J. K.; Dole, M. Diffusion in supersaturated solutions. II. Glucose solutions. *J. Am. Chem. Soc.* **1953**, *75* (16), 3900–3904. DOI: 10.1021/ja01112a008.

(7) Almond, A.; Brass, A.; Sheehan, J. K. Deducing polymeric structure from aqueous molecular dynamics simulations of oligosaccharides: predictions from simulations of hyaluronan tetrasaccharides compared with hydrodynamic and X-ray fibre diffraction data. *J. Mol. Biol.* **1998**, *284* (5), 1425–1437. DOI: https://doi.org/10.1006/jmbi.1998.2245.

(8) Fujikawa, M.; Sato, Y.; Fujita, M.; Nagasaka, Y. Mutual diffusion coefficient of concentrated trehalose aqueous solutions including supercooled regions measured by the Soret forced Rayleigh scattering method. *J. Mol. Liq.* **2020**, *311*, 113346. DOI: 10.1016/j.molliq.2020.113346.

(9) English, A. C.; Dole, M. Diffusion of sucrose in supersaturated solutions. *J. Am. Chem. Soc.* **1950**, *72* (7), 3261–3267. DOI: 10.1021/ja01163a132.

(10) Ribeiro, A. C. F.; Ortona, O.; Simões, S. M. N.; Santos, C. I. A. V.; Prazeres, P. M. R. A.; Valente, A. J. M.; Lobo, V. M. M.; Burrows, H. D. Binary mutual diffusion coefficients of aqueous solutions of sucrose, lactose, glucose, and fructose in the temperature range from (298.15 to 328.15) K. *J. Chem. Eng. Data* **2006**, *51* (5), 1836–1840. DOI: 10.1021/je0602061.

(11) Ihnat, M.; Goring, D. A. I. Shape of the cellodextrins in aqueous solution at 25 °C. *Can. J. Chem.* **1967**, *45* (20), 2353–2361. DOI: 10.1139/v67-382.

(12) Tlili, M.; Abdelmoulahi, H.; Trabelsi, S.; Nasr, S.; González, M. A.; Cuello, G. J.; Bellissent-Funel, M.-C.; Darpentigny, J. Hydrogen-bond network in liquid methanol as studied by neutron scattering, DFT calculations and molecular dynamics simulations. *J. Mol. Struct.* **2021**, *1227*, 129683. DOI: https://doi.org/10.1016/j.molstruc.2020.129683.

(13) Teychené, J.; Balmann, H. R.-d.; Maron, L.; Galier, S. Why are saccharides dehydrated in the presence of electrolytes? Insights from molecular modeling and thermodynamic measurements. *ACS Cent. Sci.* **2018**, *4* (11), 1531–1536. DOI: 10.1021/acscentsci.8b00610.

(14) Galema, S. A.; Hoeiland, H. Stereochemical aspects of hydration of carbohydrates in aqueous solutions. 3. Density and ultrasound measurements. *J. Phys. Chem.* **1991**, *95* (13), 5321–5326. DOI: 10.1021/j100166a073.

(15) Aeberhardt, K.; Laumer, J.-Y. d. S.; Bouquerand, P.-E.; Normand, V. Ultrasonic wave spectroscopy study of sugar oligomers and polysaccharides in aqueous solutions: The hydration length concept. *Int. J. Biol. Macromol.* **2005**, *36* (5), 275–282. DOI: https://doi.org/10.1016/j.ijbiomac.2005.04.006.
